# *Fuerstia marisgermanicae* gen. nov., sp. nov., an Unusual Member of the Phylum Planctomycetes from the German Wadden Sea

**DOI:** 10.3389/fmicb.2016.02079

**Published:** 2016-12-22

**Authors:** Timo Kohn, Anja Heuer, Mareike Jogler, John Vollmers, Christian Boedeker, Boyke Bunk, Patrick Rast, Daniela Borchert, Ines Glöckner, Heike M. Freese, Hans-Peter Klenk, Jörg Overmann, Anne-Kristin Kaster, Manfred Rohde, Sandra Wiegand, Christian Jogler

**Affiliations:** ^1^Leibniz Institut Deutsche Sammlung Von Mikroorganismen und ZellkulturenBraunschweig, Germany; ^2^School of Biology, Newcastle UniversityNewcastle, UK; ^3^Helmholtz Centre for Infectious DiseaseBraunschweig, Germany

**Keywords:** planctomycetes, *Fuerstia marisgermanicae*, giant genes, cell division, animal associated

## Abstract

Members of the phylum Planctomycetes are ubiquitous bacteria that dwell in aquatic and terrestrial habitats. While planctomycetal species are important players in the global carbon and nitrogen cycle, this phylum is still undersampled and only few genome sequences are available. Here we describe strain NH11^T^, a novel planctomycete obtained from a crustacean shell (Wadden Sea, Germany). The phylogenetically closest related cultivated species is *Gimesia maris*, sharing only 87% 16S rRNA sequence identity. Previous isolation attempts have mostly yielded members of the genus *Rhodopirellula* from water of the German North Sea. On the other hand, only one axenic culture of the genus *Pirellula* was obtained from a crustacean thus far. However, the 16S rRNA gene sequence of strain NH11^T^ shares only 80% sequence identity with the closest relative of both genera, *Rhodopirellula* and *Pirellula*. Thus, strain NH11^T^ is unique in terms of origin and phylogeny. While the pear to ovoid shaped cells of strain NH11^T^ are typical planctomycetal, light-, and electron microscopic observations point toward an unusual variation of cell division through budding: during the division process daughter- and mother cells are connected by an unseen thin tubular-like structure. Furthermore, the periplasmic space of strain NH11^T^ was unusually enlarged and differed from previously known planctomycetes. The complete genome of strain NH11^T^, with almost 9 Mb in size, is among the largest planctomycetal genomes sequenced thus far, but harbors only 6645 protein-coding genes. The acquisition of genomic components by horizontal gene transfer is indicated by the presence of numerous putative genomic islands. Strikingly, 45 “giant genes” were found within the genome of NH11^T^. Subsequent analysis of all available planctomycetal genomes revealed that Planctomycetes as such are especially rich in “giant genes”. Furthermore, Multilocus Sequence Analysis (MLSA) tree reconstruction support the phylogenetic distance of strain NH11^T^ from other cultivated Planctomycetes of the same phylogenetic cluster. Thus, based on our findings, we propose to classify strain NH11^T^ as *Fuerstia marisgermanicae* gen. nov., sp. nov., with the type strain NH11^T^, within the phylum Planctomycetes.

## Introduction

Planctomycetes are a phylum of ubiquitous, environmentally important bacteria that play key roles in global carbon and nitrogen cycles (Fuerst and Sagulenko, 2011; Kartal et al., 2013). Together with the Verrucomicrobia and the Chlamydia, planctomycetes belong to the PVC superphylum (Wagner and Horn, 2006). While Chlamydia are intracellular pathogens (Stephens et al., 1998), Verrucomicrobia (Petroni et al., 2000), and Planctomycetes (Fuerst et al., 1997; Lage and Bondoso, 2014) can be closely associated with eukaryotes as well. The Planctomycetes are divided into four distinct orders. While the orders Phycispherales, Tepidisphaerales, and Planctomycetales are based on axenic cultures, the order Brocadiales is formed by well described enrichment cultures, the so-called anammox-Planctomycetes. These organisms are capable of anaerobic ammonium oxidation, a trait extremely useful for wastewater treatment (Kartal et al., 2013). In particular, members of the order Planctomycetales were found to encode numerous secondary metabolite-related genes and gene clusters (Jeske et al., 2013), that were recently found to be active under conditions that chemically mimicked the interaction with eukaryotes (Jeske et al., 2016). Consequently, Planctomycetes were postulated to be “talented producers” of small molecules and they might represent a yet untapped resource of novel bioactive molecules (Jeske et al., 2016). Furthermore, the biotechnological application of planctomycetal enzymes such as sulfatases as biocatalysts was demonstrated (Wallner et al., 2005). Thus, Planctomycetes are environmentally important and of general biotechnological interest.

Planctomycetes were further proposed to comprise conspicuous cell biological features such as endocytosis-like uptake of proteins (Fuerst and Sagulenko, 2010) and compartmentalization of their cytosol (Fuerst, 2005). In addition, they were believed to lack peptidoglycan (PG) in their cell walls (König et al., 1984). Recently some of these unique features were questioned. For example, the presence of PG was demonstrated (Jeske et al., 2015; van Teeseling et al., 2015). However, in particular the unusual cell division of Planctomycetales through polar budding makes them unique among bacteria, as they lack the otherwise universal bacterial cell division protein FtsZ (van Niftrik et al., 2009; Jogler et al., 2012).

Despite their importance for environmental microbiology, biotechnology and cell biology, only 30 planctomycetal species were obtained as axenic cultures and only 10 completely closed genome sequences are available through NCBI GenBank. Thus, from a phylogenetic point of view, the phylum Planctomycetes is heavily undersampled and only few representatives of this phylum are taxonomically characterized in detail (Ward, 2010; Fuerst and Sagulenko, 2011).

In this study, we selectively target eukaryote-associated species from a marine habitat. We revisit the German North Sea, which is a well-known resource for the cultivation of Planctomycetes (Glöckner et al., 2003; Winkelmann and Harder, 2009). To focus on potentially eukaryote-associated species, we collected crab shells as the successful isolation of novel Planctomycetes from Crustacea was previously reported (Fuerst et al., 1997). As planctomycetes are known to be resistant to multiple antibiotics such as carbenicillin (Cayrou et al., 2010; Aghnatios et al., 2015), we employed a 96 well-based high-throughput cultivation procedure and subsequently screened hundreds of carbenicillin-resistant bacterial cultures with a PCR targeting the 16S rRNA gene. The obtained strain NH11^T^ was selected for further analysis and full-length 16S rRNA gene sequencing revealed its affiliation with the phylum Planctomycetes. Here we show how strain NH11^T^ differs from other planctomycetal species and therefore propose the new genus *Fuerstia* gen. nov., with the type species *Fuerstia marisgermanicae* sp. nov.

## Materials and methods

### Sampling site

The strain was isolated from a crustacean shell (crab) which was collected on the 22nd May 2012 at low tide (8:30 a.m.) at 16°C ambient temperature in some residue water on the tidal mud flat of the German Wadden Sea, Neuharlingersiel (53°42′15.5″ N, 7°42′15.9″ E).

### Isolation and maintenance

In a first step the surface of a crustacean shell was scraped-off and the shell material was transferred to sterile artificial sea water (ASW) supplemented with 10-fold diluted HD medium, to serve as biofilm suspension. ASW was prepared modified after Levring (1946) consisting of (per liter distilled water): 23.6 g NaCl; 0.64 g KCl; 4.53 g MgCl_2_ · 6 H_2_O; 5.94 g MgSO_4_ · 7 H_2_O; 1.3 g CaCl_2_ · 2 H_2_O; 10 mg Na_2_PO4 · 2 H_2_O; 2.1 mg NH_4_NO_3_. To avoid precipitation, the CaCl_2_ solution was sterilized separately (Bruns et al., 2003). HD medium was composed of 0.25 g/l yeast extract, 0.1 g/l glucose, 0.5 g/l peptone and 2.38 g/l HEPES; the pH was adjusted to 7.3. The suspension of the bacterial biofilm was used to inoculate fifty 96 well plates employing a multidrop device as previously described (Jogler et al., 2013). Cultures were subsequently transferred to fresh 96 well plates with the same medium but supplemented with 2 mg/ml carbenicillin. Cultures that survived this treatment were screened employing a 16S rRNA gene targeting PCR, using the primer set 8f (5′–AGA GTT TGA TCM TGG CTC AG–3′) and 1492r (5′–GGY TAC CTT GTT ACG ACT T–3′) modified from Lane (1991). PCR amplifications were performed employing a Veriti 96-Well Thermal Cycler (Applied Biosystems) applying the following conditions: initial denaturation at 94°C for 5 min, followed by 10 cycles of denaturation at 94°C for 30 s, annealing at 59°C for 30 s and elongation at 72°C for 60 s. This first 10 cycles were followed by 20 cycles of denaturation at 94°C for 30 s, annealing at 54°C for 30 s, elongation at 72°C for 60 s and a final elongation at 72°C for 5 min. Amplification products were subject to 16S rRNA gene sequencing and NCBI database comparison to identify novel strains. Based on this analysis, strain NH11^T^ was selected for subsequent experiments. Further cultivation was performed employing a modified M2 culture broth (M2mod) previously used for *Rhodopirellula baltica* (Jeske et al., 2013) containing 0.5 g/l peptone, 0.25 g/l glucose, 250 ml/l artificial sea water (ASW), 5 ml/l vitamin solution (double concentrated) and 20 ml/l mineral salt solution. The medium was buffered with 2.38 g/l HEPES at pH 7.0. Artificial sea water was composed of 46.94 g/l NaCl, 7.84 g/l Na_2_SO_4_, 21.28 g/l MgCl_2_ · 6 H_2_O, 2.86 g/l CaCl_2_ · 2 H_2_O, 0.384 g/l NaHCO_3_, 1.384 g/l KCl, 0.192 g/l KBr, 0.052 g/l H_3_BO_3_, 0.08 g/l SrCl_2_ · 6 H_2_O, and 0.006 g/l NaF. The vitamin solution was composed of 4 mg/l biotin, 4 mg/l folic acid, 20 mg/l pyridoxine-HCl, 10 mg/l riboflavin, 10 mg/l thiamine-HCl · 2 H_2_O, 10 mg/l nicotinamide, 10 mg/l D-Ca-pentothenate, 0.2 mg/l vitamin B_12_, and 10 mg/l *p*-aminobenzoic acid. Mineral salt solution was composed of 10 g/l nitrilotriacetic acid (NTA), 29.70 g/l MgSO_4_ · 7 H_2_O, 3.34 g/l CaCl_2_ · 2 H_2_O, 12.67 mg/l Na_2_MoO_4_ · 2 H_2_O, 99 mg/l FeSO_4_ · 7 H_2_O, and 50 ml/l metal salt sol. “44”. Metal salt sol. “44” was composed of 250 mg/l Na-EDTA, 1.095 g/l ZnSO_4_ · 7 H_2_O, 0.5 g/l FeSO_4_ · 7 H_2_O, 154 mg/l MnSO_4_ · H_2_O, 39.20 mg/l CuSO_4_ · 5 H_2_O, 24.80 mg/l Co(NO_3_)_2_ · 6 H_2_O, and 17.70 mg/l Na_2_B_4_O_7_ · 10 H_2_O. Solid medium was prepared by adding 15 g/l of three times prewashed agar (Becton, Dickinson and Company) to the medium.

### Light microscopy

Phase contrast (Phaco) and differential interference contrast (DIC) analysis were performed employing a Nikon Eclipse Ti inverted microscope with a Nikon N Plan Apochromat λ 100x/1.45 Oil objective and a Nikon DS-Ri2 camera. Specimens were immobilized in MatTek glass bottom dishes (35 mm, No. 1.5) employing a 1% agarose cushion. Images were analyzed using the Nikon NIS-Elements software (Version V4.3).

### Electron microscopy

For field emission scanning electron microscopy (FESEM) bacteria were fixed in 1% formaldehyde in HEPES buffer (3 mM HEPES, 0.3 mM CaCl_2_, 0.3 mM MgCl_2_, 2.7 mM sucrose, pH 6.9) for 1 h on ice and washed one time employing the same buffer. Cover slips with a diameter of 12 mm were coated with a poly–L–lysine solution (Sigma–Aldrich) for 10 min, washed in distilled water and air–dried. Fifty microliter of the fixed bacteria solution was placed on a cover slip and allowed to settle for 10 min. Cover slips were then fixed in 1% glutaraldehyde in TE buffer (20 mM TRIS, 1 mM EDTA, pH 6.9) for 5 min at room temperature and subsequently washed twice with TE–buffer before dehydrating in a graded series of acetone (10, 30, 50, 70, 90, and 100%) on ice for 10 min at each concentration. Samples from the 100% acetone step were brought to room temperature before placing them in fresh 100% acetone. Samples were then subjected to critical–point drying with liquid CO_2_ (CPD 300, Leica). Dried samples were covered with a gold/palladium (80/20) film by sputter coating (SCD 500, Bal–Tec) before examination in a field emission scanning electron microscope (Zeiss Merlin) using the Everhart Thornley HESE2–detector and the inlens SE–detector in a 25:75 ratio at an acceleration voltage of 5 kV. TEM micrographs of NH11^T^ cells were taken after negative staining with aqueous 0.1–2% uranyl acetate, employing a Zeiss transmission electron microscope EM 910 at an acceleration voltage of 80 kV at calibrated magnification as previously described (Wittmann et al., 2014). Prior TEM analysis cells were fixated for 2 h with 3% glutaraldehyde in 3 mM EM-HEPES buffer.

Thin sections of strain NH11^T^ were prepared by high pressure freezing and freeze substitution as previously described (Jogler et al., 2011). Sections were subsequently analyzed employing a JEOL 1200EX–80kV TEM microscope.

### Physiological tests

Physiological tests such as salinity, pH, and temperature tolerance were performed in liquid medium M2mod. To test the NaCl tolerance, medium M2mod was prepared employing ASW devoid of NaCl. The required NaCl concentrations were adjusted prior inoculation, using a 30% NaCl (w/v) solution. The ASW tolerance was tested using M2mod without ASW. Again, the required concentration of ASW was adjusted prior inoculation. Growth was detected by monitoring the optical density at 600 nm using a Photometer Ultrospec II (LKB Biochrom). Carbon source utilization was tested using the GN2 MicroPlate™ system (Biolog), while enzymatic activities were tested using the API® ZYM method (bioMérieux). The physical features of the cell wall were analyzed by Gram staining, KOH test and Bactident® Aminopeptidase test strips. Growth under anoxic conditions was investigated using the api® 20 NE system (bioMérieux) according to the manufacturer's instructions.

### Phylogenetic analysis and tree reconstruction

For phylogenetic analysis version 6.0.2 of the ARB software package (Ludwig et al., 2004) was used together with the SILVA database SSURef_NR99 (Version 119, released on 14.07.2014; Pruesse et al., 2007). The full length 16S rRNA gene sequence was imported into ARB and then aligned using the fast aligner tool of the ARB software package. The resulting alignment was further edited manually to improve alignment quality. During the phylogenetic tree reconstruction, different type strains of the phylum Planctomycetes were used as reference sequences, while type strains of the phylum Verrucomicrobia served as out-group. All sequences that were included are listed in Table S1. Tree reconstruction was performed with the ARB software package (Ludwig et al., 2004). The Maximum Likelihood RAxML module was used with the rate distribution model GTR GAMMA running the rapid bootstrap analysis algorithm. The Neighbor Joining tool was employed with Felsenstein correction, while the Maximum Parsimony analysis was achieved with the Phylip DNAPARS module. Bootstrap values for all three methods were calculated with 1000 resamplings including the *E. coli* 16S rRNA gene positions 101–1371.

To facilitate the taxonomic classification of strain NH11^T^, a cluster analysis was performed employing version 6.0.6 of the ARB software package (Ludwig et al., 2004) together with the database SSURef_NR99 (Version 123.1, released on 03.03.16) using a 87.65% sequence identity cutoff (threshold for the minimal sequence identity within a taxonomic family after Yarza et al., 2014) and *E. coli* 16S rRNA gene positions 112–1393. This analysis included all sequences of the family *Planctomycetaceae* that were available in the database as well as sequences of uncultivated planctomycetes, while the three clusters obtained for *Rubinisphaera brasiliensis* DSM 5305^T^, *Gimesia maris* 534-30^T^ and strain NH11^T^ were selected for further phylogenetic tree reconstruction. For the NH11^T^ containing cluster, the highest, lowest, average and median sequence identities were determined by calculating a distance matrix based on the *E. coli* 16S rRNA position 112–1393.

### Genome sequencing

Genomic DNA of strain NH11^T^ was extracted using the Genomic-tip 20/G kit (Qiagen, Germany) and a >10 kb SMRTbell™ template library was prepared according to the manufacturer instructions (PacificBiosciences, USA). In brief, ~10 μg of genomic DNA was end-repaired and ligated to hairpin adapters overnight (DNA/Polymerase Binding Kit 2.0, Pacific Biosciences, USA). The SMRTbell™ template was exonuclease treated for removal of incompletely formed reaction products. Conditions for annealing of sequencing primers and binding of polymerase to purified SMRTbell™ template were assessed with the calculator in RS Remote (PacificBiosciences, USA). Five SMRT cells were sequenced on the PacBio RS/RSII (PacificBiosciences, USA), each covered with a 90-min movie. Eight additional SMRT cells were used applying the DNA/Polymerase Binding Kit P4 (PacificBiosciences, USA) in order to collect larger read lengths. For those cells, 180-min movies were taken and a sub read lengths up to 20 kb was observed.

In addition, 2 × 9,143,110 paired-end Illumina reads were obtained employing an Illumina HiSeq 2500 for 101 cycles in both directions using the TruSeq DNA Sample Prep Kit v2 according to the manufacturer's instructions (Illumina Inc., San Diego, CA, USA). The genome assembly was performed using the RS_HGAP_Assembly.2 protocol included in SMRT Portal version 2.2.0 utilizing 520,016 reads from all 13 SMRT cells applying standard parameters with exception of the genome size, which was set to 9,100,000. Thus, one final contig could be obtained, which afterwards was error-corrected using a subset of 2 × 4,000,000 Illumina reads using BWA (Li and Durbin, 2009) with subsequent variant and consensus calling employing the CLC Genomics Workbench 7.03 (http://www.clcbio.com). Visual inspection of 41 called variants has been performed using IGV (Thorvaldsdóttir et al., 2013) in addition to prediction of rRNAs using RNAmmer (Lagesen et al., 2007). Hereby, the error-corrected consensus was trimmed, circularized and adjusted to *dnaA* as the first gene. For the circular genome of *Fuerstia marisgermanicae* NH11^T^ the finishing quality is estimated to be at 99.9999% (QV 60) confirmed by final PacBio resequencing. Genome annotation has been performed using PROKKA 1.8 (Seemann, 2014).

### Genome analysis

Genomic islands and CRISPR regions were identified using IslandViewer3 (Dhillon et al., 2015) and CRT (Bland et al., 2007), respectively. The planctomycetal genomes for the gene content analysis were derived from NCBI and IMG (Markowitz et al., 2012) in April 2016 and had to match the following criteria upon CheckM analysis (Parks et al., 2015): completeness >90, contamination <5 and strain heterogeneity <20. Orthologs were detected with Proteinortho5 (Lechner et al., 2011), a tool that identifies the reciprocal best hits from the given protein sequences. The genome plot was then generated with BRIG (Alikhan et al., 2011). The G+C content of the DNA was estimated by analyzing the genome data using the software Artemis (Rutherford et al., 2000).

The gene size was calculated from annotations and analyzed with R (R Core Team, 2015) using the packages ggplot2 (Wickham, 2009), reshape (Wickham, 2007), xlsx (Dragulescu, 2014). Threshold for large genes were set to 5 kb according to Reva and Tümmler (2008). The biggest giant genes of NH11^T^ with a size over 20 kb were further analyzed using the InterProScan web service (Jones et al., 2014; Mitchell et al., 2015). The genome of strain NH11^T^ was screened for putative secondary metabolite clusters using the tool antiSMASH 3.0 (Weber et al., 2015).

### Multilocus sequence analysis (MLSA)

Orthologs were identified using Proteinortho5 (Lechner et al., 2011) with the “-selfblast” option (which enables paralog-detection) enabled. Only genes present exclusively in single copy in all compared genomes were selected for MLSA. Alignments of the respective gene product amino acid sequences were generated individually for each ortholog group using MUSCLE v3.8.31 (Edgar, 2004a,b) and subsequently concatenated. Unalignable regions, caused by e.g., unique N-terminal or C-terminal sequence overhangs, were filtered from the concatenated alignment using Gblocks v.0.91b (Castresana, 2000). Phylogenetic relationships were inferred from the remaining unambiguous alignment positions by Neighbor Joining clustering with 1000 bootstrap iterations, using ARB 6.0.5 (Ludwig et al., 2004) and by Maximum Likelihood calculation using RAxML v. 8.0.26 (Stamatakis, 2014).

### Fatty acid analysis

For fatty acid analysis, biomass of the three compared strains (NH11^T^, *R. brasiliensis* DSM 5305^T^ and *G. maris* 534-30^T^) was obtained from liquid cultures, grown in M2mod culture broth, at 20°C and slight agitation in baffled flasks. The obtained biomasses were processed according to the standards of the Identification Service of the German Collection of Microorganisms and Cell Cultures (DSMZ) (Miller, 1982; Kuykendall et al., 1988; Kämpfer and Kroppenstedt, 1996).

### Nucleotide sequence accession numbers

The accession numbers of the nucleotide sequences used for giant gene analysis or phylogenetic reconstruction are shown in Table S1. The NCBI genome accession number is CP017641.

## Results

### Phylogenetic analysis

**Figure 1:**
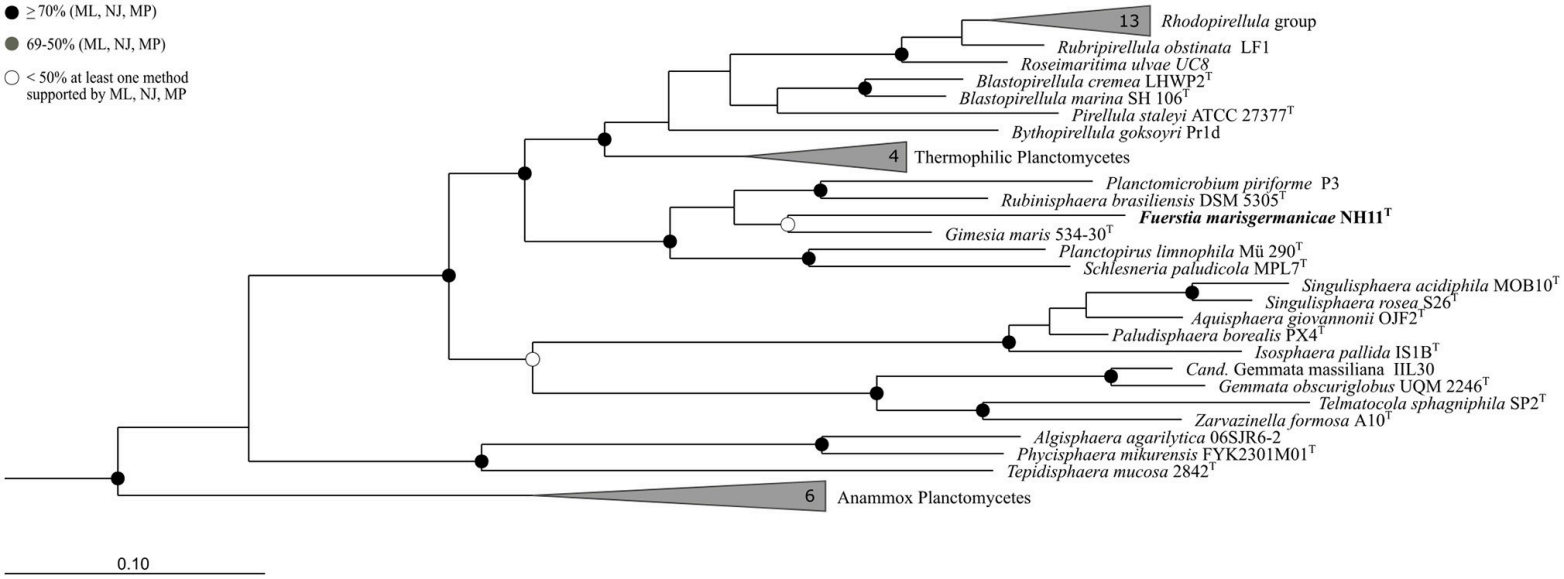
**Phylogeny of strain NH11^**T**^**. A maximum-likelihood 16S rRNA gene based tree of the phylogenetic position of strain NH11^T^. Strain NH11^T^ is highlighted in bold letters and in comparison planctomycetal type strains are shown, while selected Verrucomicrobia served as outgroup. The bootstrap percentages of 1000 resamplings of three different tree building methods (ML, Maximum Likelihood; NJ, Neighbor Joining; MP, Maximum Parsimony) are incorporated in this tree, indicated by different shaded dots. Black dots indicate support values above 70% for all three methods while white dots refer to a bootstrap support below 50% for at least one method. Branches that were not supported by all three methods show no dot.

Based on 16S rRNA gene phylogeny (Figure 1), strain NH11^T^ clusters together with *G. maris* within the family *Planctomycetaceae* (bootstrap support values: 69% for Maximum Likelihood, 43% for Maximum Parsimony, 46% for Neighbor Joining). However, it shares only 87.1% 16S rRNA gene sequence identity with *G. maris* based on BLAST analysis. Its next closest relative besides *G. maris* is *R. brasiliensis* (84.5% 16S rRNA gene sequence identity). An overview of the identities, generated with the manually curated SILVA alignment of all species of the phylum Planctomycetes, based on the 1270 base pairs used for phylogenetic reconstruction, is given in Table S2. In this analysis the Felsenstein correction was used, to take evolutionary events that occurred during speciation into account. According to this analysis strain NH11^T^ shares 85.4% identity with *G. maris* and 84.1% with *R. brasiliensis*, differing from BLAST search results described above. The performed cluster analysis revealed, that NH11^T^, *G. maris* and *R. brasiliensis* form three distinct clusters within the family *Planctomycetaceae* with an identity of at least 87.65% within each cluster, which is identical with the minimum sequence identity threshold for taxonomic families (Yarza et al., 2014; Figures S1–S3). In particular, within the cluster of strain NH11^T^ the minimal sequence identity is 88.3% and the maximal identity is 99.9%. The average identity in this cluster is 94.48% with a median of 94.4%, matching the threshold of 92.25% for median sequence identity for taxonomic families.

### Morphological characterization

**Figure 2:**
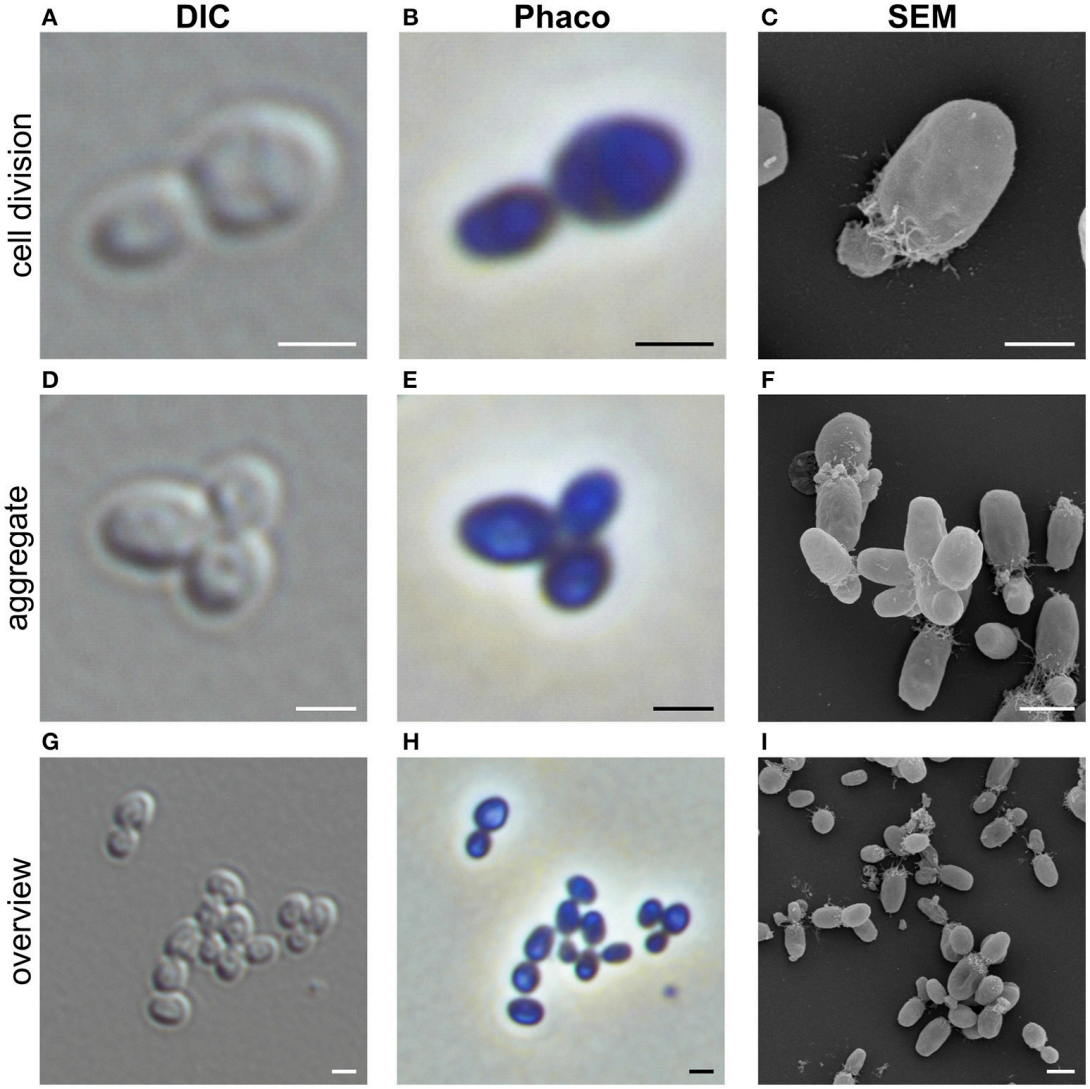
**Morphology of strain NH11^**T**^**. Representative differential interference contrast (DIC), Phase-contrast (Phaco) and scanning electron microscopic (SEM) micrographs of strain NH1. Shown are individual representative cells during division through polar budding **(A–C)** and -aggregate formation **(D–F)**. In addition, an overview of multiple cells from a representative liquid culture is provided **(G–I)**. Bar, 1 μm.

**Figure 3:**
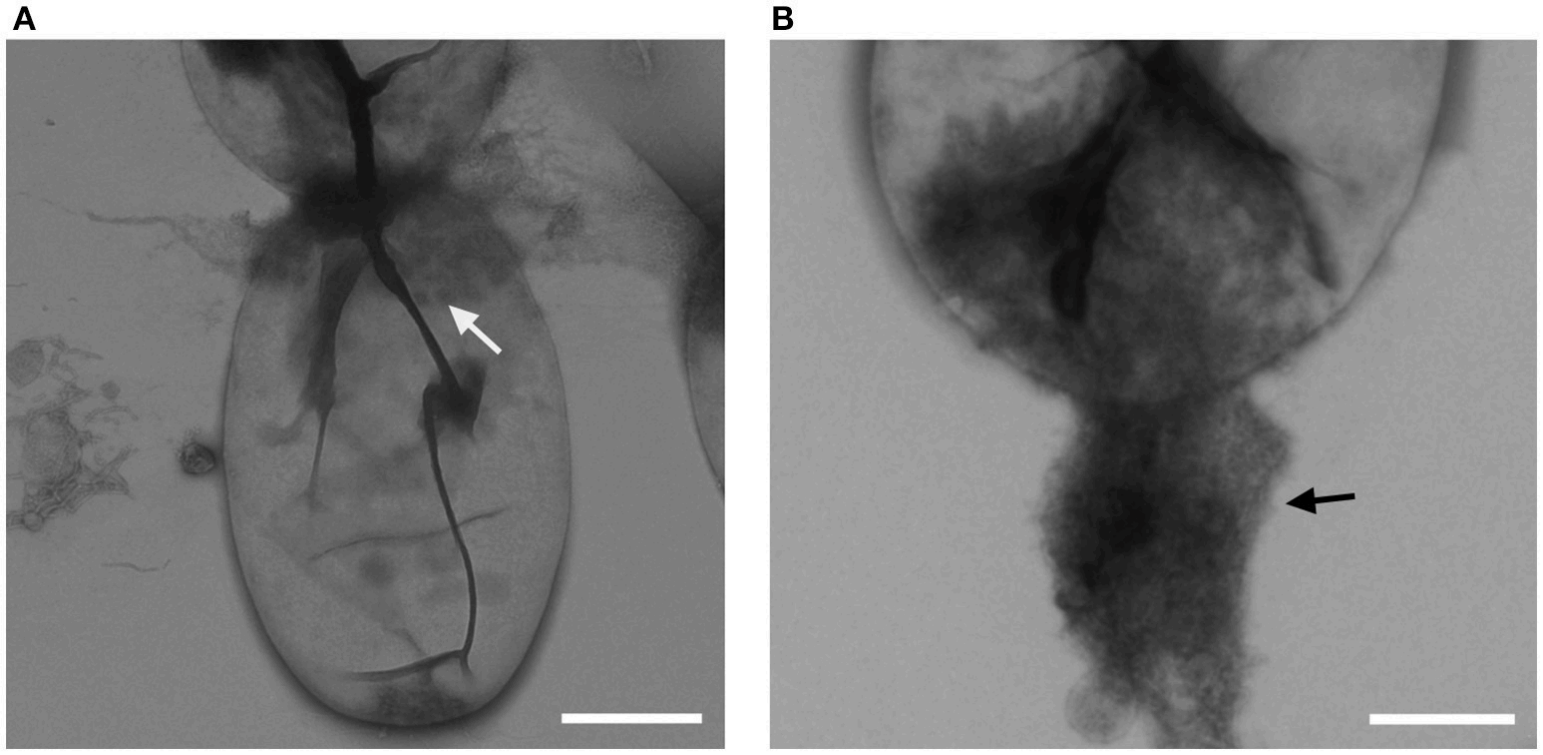
**Crateriform structures of strain NH11^**T**^**. TEM analysis of negative stained NH11^T^ cells revealed crateriform structures solely on the cell pole (**A**, white arrow) and fiber like structures seem to arise from such pits (**B**, black arrow). Bar, 0.5 μm.

**Figure 4:**
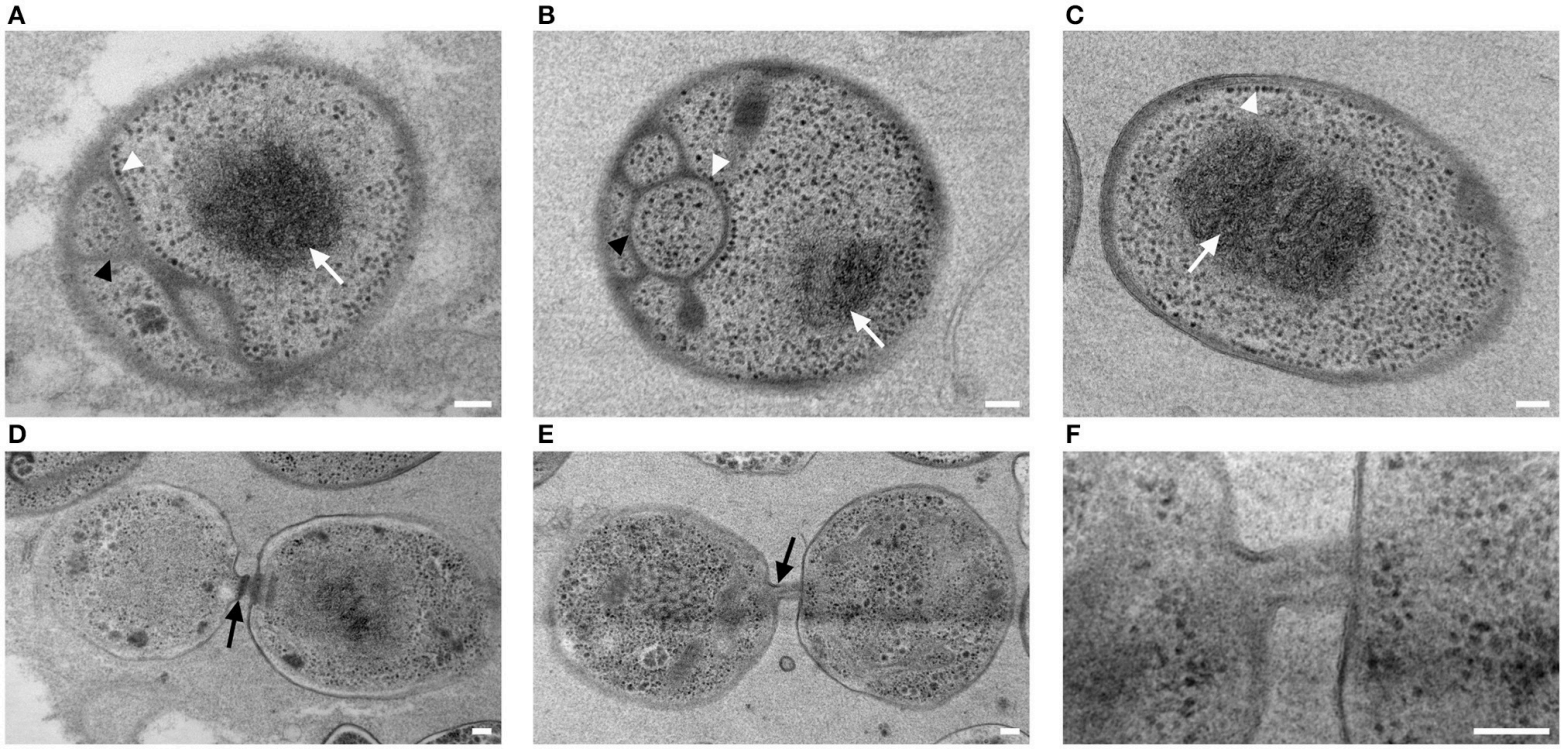
**Subcellular organization and cell division of strain NH11^**T**^**. High pressure frozen and freeze substituted cells of strain NH11^T^ were subject to thin-sectioning and TEM analysis. Three representative sections of individual cells correspond to differences in subcellular organization of strain NH11^T^ **(A–C)**. Cells always comprise a condensed nucleoid which is visible as a black structure (white arrow), while their cytoplasmic membrane can show different degrees of invaginations (**A,B**, black arrowheads). Some cells even lack any invagination of the cytoplasmic membrane **(C)**. Some ribosomes (white arrowheads) are always located at the cytoplasmic membrane despite its degree of invagination **(A–C)**. Dividing cells are interconnected by a tubular like structure **(D–F)**. Between mother and daughter cell, sometimes a black structure, potentially correlating with a division ring, became visible (**D**, black arrow). This structure could only be observed in some sections, while it was absent in others **(E–F)**. Bar, 0.1 μm.

Cells of strain NH11^T^ are pear to ovoid shaped (Figure 2) and form cream colored colonies on solid medium and motile swimmer cells in liquid culture. Cells are 1.2–2.5 × 0.9–1.7 μm in size. Its major phenotypic characteristics compared to those of *G. maris* and *R. brasiliensis* are listed in Table 1. No rosettes or stalks are formed in culture in contrast to the closest cultivated relatives *G. maris* and *R. brasiliensis* (Figures 2, 3). The surface of strain NH11^T^ is smooth and seems to lack crateriform structures except of polar regions were fiber-like structures seem to emerge from crateriform pits (Figure 3). Furthermore, the cell architecture of strain NH11^T^ and its mode of division seem to differ from all other planctomycetes described thus far (Figure 2). High pressure frozen and freeze substitution sections of strain NH11^T^ display a condensed nucleoid that was previously described in other planctomycetes, too (Figures 4A–C, white arrow). However, some cells display exceptional patterns of cytoplasmic invaginations (Figures 4A,B), while others, except for the condensed nucleoid, comprise a cell envelope comparable to *E. coli* (Figure 4C). Despite of the degree of invaginations with characteristics different from other planctomycetes, ribosomes seem to localize frequently in close proximity to the cytoplasmic membrane in a rope-of-pearls-like pattern (Figures 4A–C, white arrowheads). Cells of strain NH11^T^ divide through budding and unlike all other planctomycetes described thus far, the mother- and daughter-cells seem to be connected via a tubular structure during cell division (Figures 4D–F). In particular, a dark structure at the division plane seems to parallel the cell division ring previously described for anammox Planctomycetes (van Niftrik et al., 2009; Figure 4D, black arrow).

**Table 1:** **Morphological- and physiological features**.

**Characteristic**	**NH11^T^**	***G. maris***	***R. brasiliensis***
Cell shape	Pear shaped to ovoid	Spherical to ovoid	Spherical to ovoid
Cell size, μm	1.2–2.5 × 0.9–1.7	0.4–1.5	0.7–1.8
Flagellation	+	+	+
Rosette formation	−	+	+
Stalk formation	−	+	+
Colony color	Cream	Cream	Yellow to ochre
ASW tolerance (%)	27.5–230	25–150	20–300
ASW optimum (%)	50–117.5	n.d.	40–180
NaCl tolerance (%, w/v)	<5	1.5-4	0.6–10
pH growth range	6–10	n.d.	n.d.
pH optimum	7	7	7.5
Temperature range, °C	20–30	6–37	<38
Temperature optimum, °C	28	30	27–35

*Comparison of morphological- and physiological features of strain NH11^T^ with the two closest related type species Gimesia maris (Bauld and Staley, 1976) and Rubinisphaera brasiliensis (Schlesner, 1989)*.

### Physiological characterization

Strain NH11^T^ is able to grow between pH 6 and 10, with an optimal growth at pH 7 (Figure S4A). NH11^T^ tolerates up to 5% NaCl supplementation (Figure S4B) and requires at least 27.5% ASW for detectable growth (Figure S4C). In contrast, up to 230% ASW can be tolerated, while optimal growth conditions are in the range of 50–117.5% ASW. Temperature-wise, strain NH11^T^ can grow between 20 and 30°C, with optimal growth at 28°C (Figure S4D). Thus, strain NH11^T^ is a mesophilic organism. It is capable of utilizing a variety of carbon sources, listed in Table 2. In particular N-acetyl-D-galactosamine, N-acetyl-D-glucosamine, L-arabinose, D-cellobiose, D-galactose, gentiobiose, α-D-glucose, α-D-lactose, lactulose, maltose, D-mannose, D-melibiose, β-methyl-D-glucoside, sucrose, D-trehalose, turanose, succinic acid mono-methyl-ester, acetic acid, γ-hydroxybutyric acid, itaconic acid, propionic acid and glycerol were utilized. In contrast to the closest related *G. maris* and *R. brasiliensis*, strain NH11^T^ was not able to use L-rhamnose as carbon source. The enzymatic repertoire of strain NH11^T^ comprises alkaline phosphatase, esterase, esterase lipase, leucinearylamidase, valinearylamidase, cysteine arylamidase, trypsin, α-chymotrypsin, acid phosphatase, naphthol-AS-BI-phosphohydrolase, α-galactosidase, and α-glucosidase. All enzymatic features are listed in Table 3. No growth under anoxic conditions was observed.

**Table 2:** **Carbon sources utilized by strain NH11^**T**^**.

**Carbon sources**	
N-Acetyl-D-Galactosamine	+
N-Acetyl-D-Glucosamine	+
L-Arabinose	+
D-Cellobiose	+
D-Galactose	+
Gentiobiose	+
α-D-Glucose	+
α-D-Lactose	+
Lactulose	+
Maltose	+
D-Mannose	+
D-Melibiose	+
β-Methyl-D-Glucoside	+
L-Rhamnose	−
Sucrose	+
D-Trehalose	+
Turanose	+
Succinic Acid Mono-Methyl-Ester	+
Acetic Acid	+
D-Glucuronic Acid	+
γ-Hydroxybutyric Acid	W
Itaconic Acid	W
Propionic Acid	W
Glycerol	+

*+, positive; −, negative; W, weakly positive*.

**Table 3:** **Enzymatic activities of strain NH11^**T**^, determined with the API ZYM test**.

**Enzymatic activities**	
Alcaline phosphatase	+
Esterase (C 4)	+
Esterase lipase (C 8)	+
Lipase (C 14)	−
Leucinearylamidase	+
Valinearylamidase	+
Cysteine arylamidase	+
Trypsin	+
α-chymotrypsin	+
Acid phosphatase	+
Naphthol-AS-BI-phosphohydrolase	+
α-galactosidase	−
β-galactosidase	−
β-glucuronidase	−
α-glucosidase	+
β-glucosidase	−
N-acetyl-β-glucosaminidase	−
α-mannosidase	−
α-fucosidase	−

*+, positive; −, negative*.

### Lipid composition

The fatty acids of strain NH11^T^ and its closest relatives *G. maris* and *R. brasiliensis* were analyzed and compared. The major fatty acids of strain NH11^T^ consist of 59.16% C_16:1_ ω6c_/16:1_ ω7c (summed feature 3), 19.83% C_18:1_ ω6c/_18:1_ ω7c (summed feature 8) and 15.12% C_16:0_. In comparison, *G. maris* comprised 26.63% of C_16:1_ω6c/_16:1_ ω7c (summed feature 3), 23.50% of C_16:0_, and 12.98% of C_16:0_ 10-methyl/Iso-C_17:1_ ω6c (summed feature 9). The fatty acid profile of *R. brasiliensis* is composed of 47.10% C_16:0_ and 45.77% C_16:1_ω6c/_16:1_ ω7c (summed feature 3). The complete fatty acid profiles of strain NH11^T^, *G. maris* and *R. brasiliensis* are listed in Table 4.

**Table 4:** **Cellular fatty acid contents (%) of NH11^**T**^ in comparison to ***G. maris*** and ***R. brasiliensis*****.

**Fatty acid**	**NH11^T^**	***G. maris***	***R. brasiliensis***
**SATURATED**			
C_14:0_	0.18	0.32	0.27
C_16:0_	15.12	23.50	45.77
C_17:0_	0.44	7.11	0.53
C_17:0_ 10-methyl	–	1.03	–
C_18:0_	0.61	1.41	0.56
**UNSATURATED**			
C_15:1_ω6c	0.88	2.46	0.28
C_16:1_ω5c	0.45	0.36	0.36
C_17:1_ω6c	1.82	4.63	–
C_17:1_ω7c	0.62	–	–
C_17:1_ω8c	–	3.05	0.30
C_18:1_ω5c	0.14	–	–
C_18:1_ω7c	–	5.76	–
C_18:1_ω9c	–	2.43	1.02
C_20:1_ω7c	–	0.26	2.76
**BRANCHED**			
Iso-C_16:0_	0.19	1.96	–
**3-HYDROXY**			
C_12:0_ 3-OH	–	0.33	–
**SUMMED FEATURES**			
2: C_14:0_ 3-OH/Iso-C_16:1_	0.55	–	–
3: C_16:1_ω6c/_16:1_ ω7c	59.16	26.63	47.10
8: C_18:1_ω6c/_18:1_ ω7c	19.83	5.76	1.06
9: C_16:0_ 10-methyl/Iso-C_17:1_ω6c	–	12.98	–

*Major fatty acids of the strain NH11^T^ are C_16:1_ω6c/_16:1_ ω7c (Summed feature), C_18:1_ω6c/_18:1_ ω7c (Summed feature) and C_16:0_*.

Taken together, the general fatty acid profile of strain NH11^T^ is similar, yet distinct compared to its closest relatives *G. maris* and *R. brasiliensis* and differs mainly in the components proportions.

### Genome analysis

**Figure 5:**
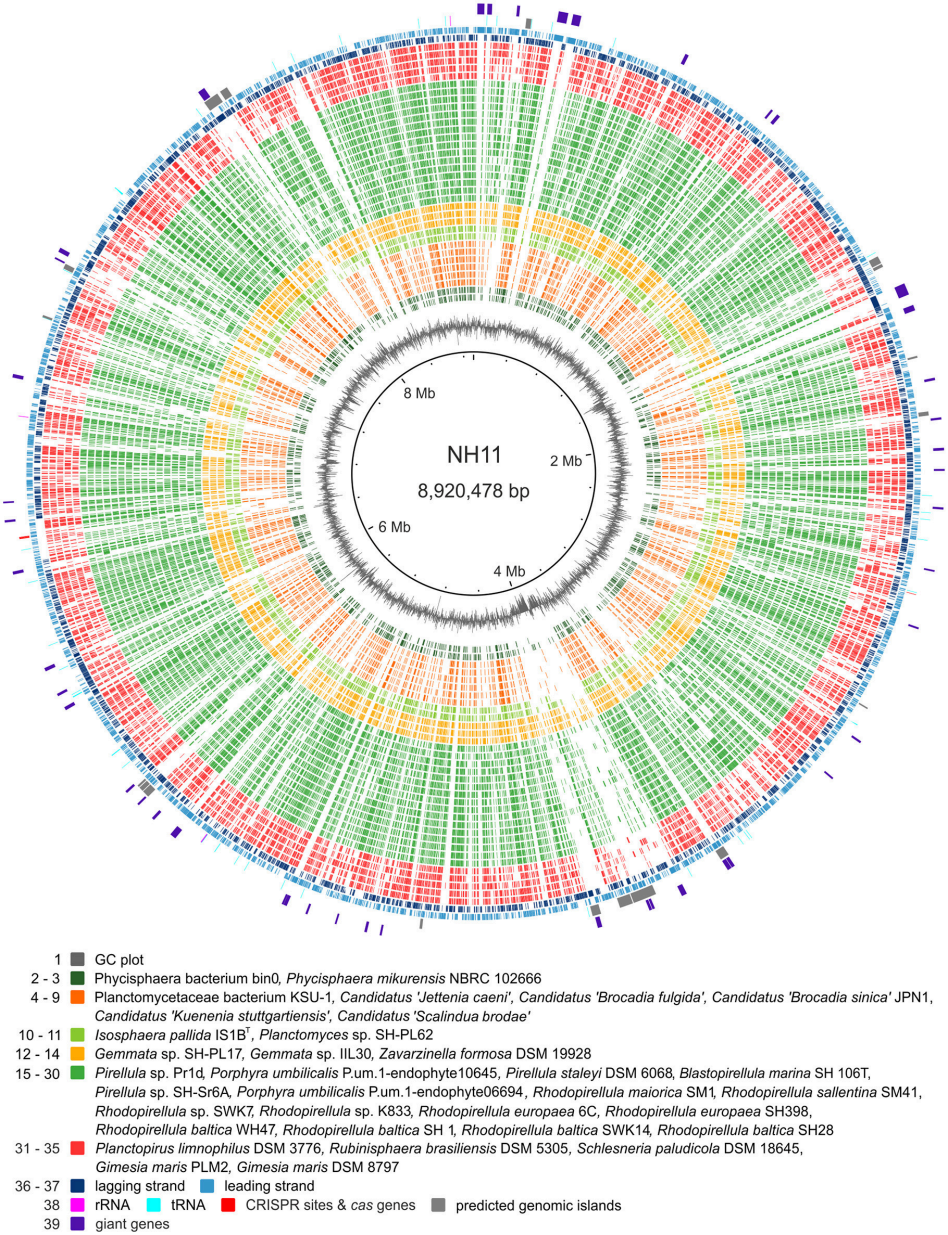
**Genomic features of strain NH11^**T**^**. The circular plot of strain NH11^T^'s 8.9 Mb chromosome summarizes multiple genomic features: the outer circles display protein (light and dark blue), tRNA (turquoise), and rRNA (pink) encoding genes as well as predicted genomic islands (gray) and giant genes (purple). The innermost circle shows the GC plot (gray). In between, ortholog genes from planctomycetal strains, for which high quality genomes were available, were identified by reciprocal BLAST and are depicted in green, yellow, and orange, according to the color scheme of Figure 7. Notable islands of unique gene content in NH11^T^ are visualized as gaps and they are found to in regions of predicted genomic islands and/or giant genes.

**Figure 6:**
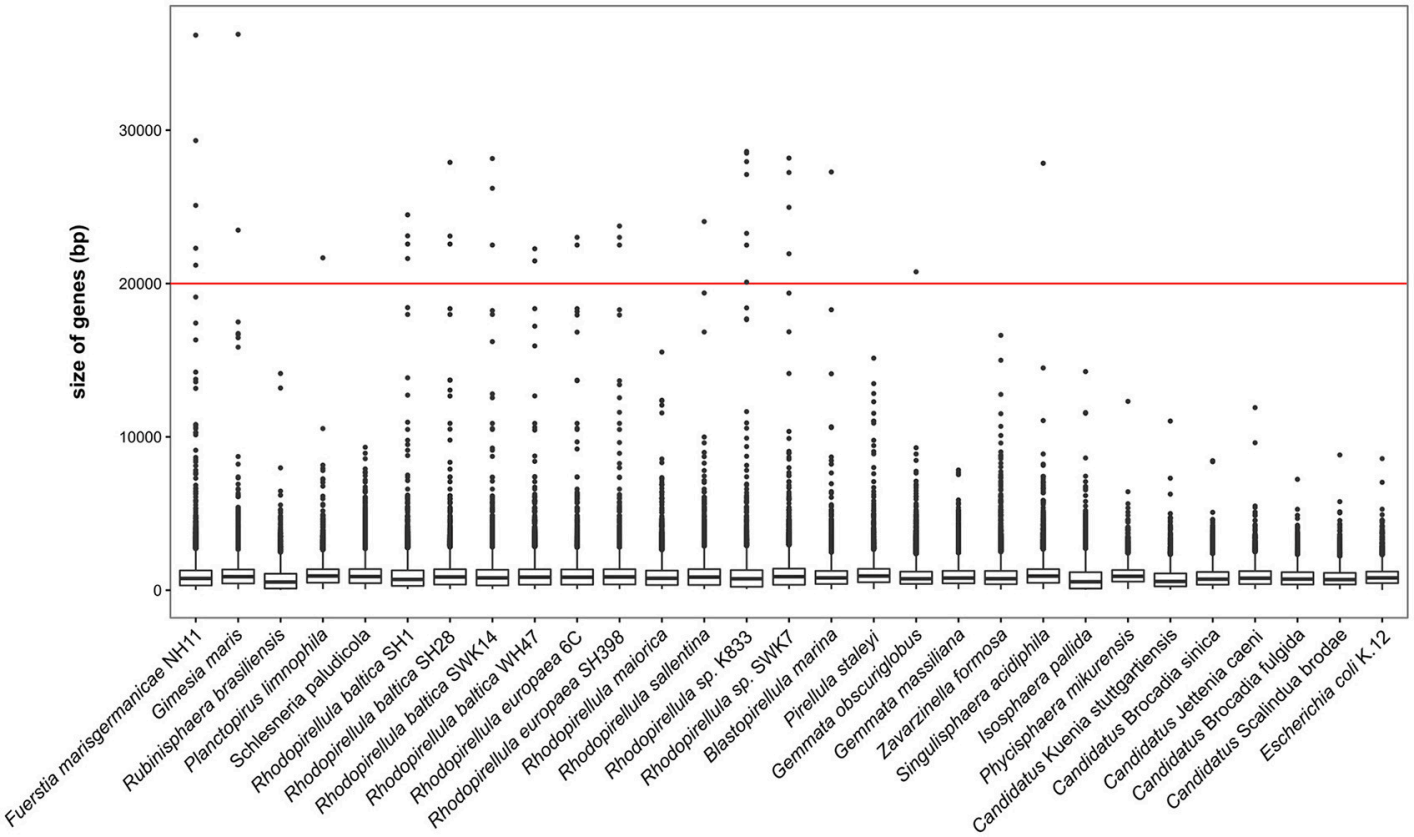
**Planctomycetal giant genes**. The gene size distribution of different Planctomycetes with available genomes is ordered by declining phylogenetic relationship to NH11^T^. Median size of genes, 25 and 75% percentiles are shown and whiskers represents the 1.5^*^ interquartile range and dots outliers. *Fuerstia marisgermanicae* comprises 45 giant genes (>5 kb), the biggest gene is 36,239 bp in size.

The complete chromosome of strain NH11^T^ comprises 8,920,478 bp, with a GC content of 55.9%. Within the genome, 6732 genes were annotated, of which 6645 were identified as protein-coding genes. As known for other planctomycetes (Fuerst and Sagulenko, 2011), only for 54.3% of these genes a function could be predicted, while the remaining genes were annotated as hypothetical proteins or proteins with unknown function. Notable islands of unique gene content were found to especially comprise regions of predicted genomic islands and regions containing so-called “giant genes” (Reva and Tümmler, 2008). The genomic features of strain NH11^T^ are summarized in Figure 5. In total, the genome of strain NH11^T^ comprises 45 giant genes, with a size greater than 5 kb (Figure S5). By the time of the first description of giant genes in the genome of *R. baltica* (Reva and Tümmler, 2008) no other planctomycetal genome sequence was available. Thus, we analyzed the general distribution of giant genes amongst Planctomycetes and found all 29 available planctomycetal genomes to encode such genes (Figure 6). Fifteen strains encoded genes with a size >20 kb, two of which *Rhodopirellula* sp. K833 and NH11^T^, even encode five >20 kb genes (Figure S6). Thus, genes >20 kb could be found in less than 50% of the available planctomycetal genomes. Furthermore, genes >30 kb were exclusively found in strain NH11^T^ and *G. maris*. Thus strain NH11^T^ is somewhat unique if gene-length is compared to other planctomycetes. Consequently, we analyzed the potential function of five NH11^T^ protein-coding genes >20 kb. The results, obtained from the InterProScan web service, points toward potential functions in cell adhesion, carbohydrate binding and partial location on the outer membrane of NH11^T^.

Because TEM analysis pointed toward an anammox Planctomycetes-like cell division ring (van Niftrik et al., 2009; Figure 4D, black arrow), the genome was analyzed toward the presence of the putative cell division ring protein of *Candidatus* “K. stuttgartiensis” (locus tag kustd1438, NCBI Accession Number CAJ72183). The best match was a hypothetical protein with 36% sequence identity but only 19% query coverage (Fuma_00096), suggesting that this is presumably not a homolog of the putative protein found in *Candidatus* “K. stuttgartiensis.” However, with a size of 8699 bp, the gene that encodes for this hypothetical protein of strain NH11^T^ belongs to the identified giant genes. Analysis of this hypothetical protein, using the InterProScan web service, revealed the presence of Lamin Tail- (IPR001322), Ig-like- (IPR032812), and PapD-like domains (IPR008962), indicating putative involvement of this protein in cell shape maintenance or sub cellular organization.

Besides the giant genes, further analysis of the genome revealed the presence of a complete CRISPR type II system (cas9: Fuma_04885, cas1: Fuma_4887, cas2: Fuma_4888; Taylor et al., 2015). Only 2 other of the 35 analyzed planctomycetal genomes, corresponding to organisms *Blastopirellula marina* SH 106 and *Candidatus* “Brocadia sinica JPN1,” comprise such a system while the closest relative of strain NH11^T^, *G. maris* lacks equivalent genes. This indicates that bacteriophages can infect strain NH11^T^ as the CRISPR type II system is primarily used for phage defense. Furthermore, absence in *G. maris* indicates differences in terms of phage susceptibility and/ or defense strategy in comparison to strain NH11^T^.

As Planctomycetes are a known source of potential bioactive compounds (Jeske et al., 2013), the genome of strain NH11^T^ was analyzed using antiSMASH 3.0 (Weber et al., 2015). In total, four secondary metabolite-associated gene clusters were found. In contrast, *G. maris*, as closest relative to NH11^T^, encodes 9 putative secondary metabolite related genes and gene clusters. Thus, again, strain NH11^T^ differes from its peers.

However, the four identified genes and clusters might be related to bacteriocins (2), terpenes (1), and ectoines (1) based on the antiSMASH prediction. Ectoins are compatible solutes, playing a role in the salt stress response, e.g., in halophilic eubacteria (Peters et al., 1990; Bernard et al., 1993; Pastor et al., 2010). However, physiological tests gave no evidence for an increased salt tolerance [>5% NaCl (w/v)] of strain NH11^T^ (see Table 1).

**Figure 7:**
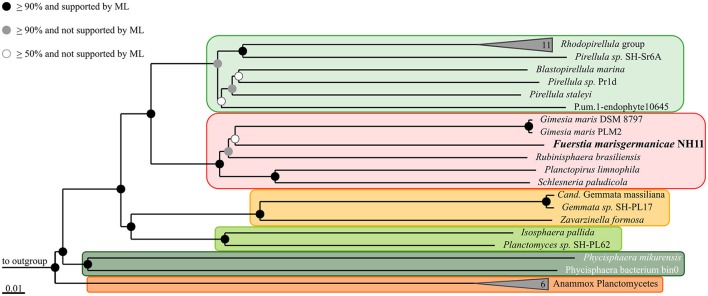
**Multilocus sequence analysis (MLSA) of strain NH11^**T**^**. The phylogenetic tree is based on Neighbor Joining analysis, with bootstrap values (presented by black, gray and white dots) based on 1000 permutations. Additionally, Maximum Likelihood (ML) analysis was performed and is also indicated by dots. The performed calculations where based on 68,695 unambiguous amino acid sequence alignment positions of 143 concatenated orthologous single-copy protein gene products shared by all 37 included genomes. The ortholog selection was based on bidirectional BLAST analysis as implemented in Proteinortho5 (Lechner et al., 2011). Sequences were aligned individually for each ortholog group and subsequently concatenated. Unalignable regions were filtered from the alignments using Gblocks (Castresana, 2000). The genomes of *Opitutus terrae* PB90-1 and *Verrucomicrobium spinosum* DSM 4136 served as outgroup. To enhance clarity, several monophyletic groups of reference organisms are shown collapsed as others are indicated by orange and green boxes, according to the color scheme of Figure 5.

The genome sequence of strain NH11^T^ was further used to verify the 16S rRNA gene sequence based phylogeny via Multi Locus Sequence Analysis (MLSA). This method enables to increase the phylogenetic resolution and to avoid the bias caused by single marker gene-based phylogenies. This was achieved by considering sequences of multiple independent single copy genes shared by the comparison organisms (Glaeser and Kämpfer, 2015). The MLSA-based phylogeny is shown in Figure 7 and further supports our 16S rRNA gene based placement.

## Discussion

Strain NH11^T^ is in many ways an unusual planctomycete. Based on 16S rRNA gene sequence comparison, it branches within the former genus *Planctomyces* which was recently found to be polyphyletic. Consequently, it was further subdivided into the separate genera *Gimesia, Planctopirus, Rubinisphaera*, and *Planctomyces*, which all belong to the order Planctomycetales and the family *Planctomycetaceae* (Scheuner et al., 2014). Similar results were obtained by a MLSA phylogenetic analysis, while both approaches provided rather low bootstrap support. However, since both methods gave similar results, strain NH11^T^ is likely to cluster within the family *Planctomycetaceae*.

To allow a detailed taxonomic classification, we performed further 16S rRNA gene sequence comparison (Table 1). We found, if evolutionary events were considered, that strain NH11^T^ belongs to a novel family based on current thresholds (Yarza et al., 2014). In contrast, direct comparison, without acknowledging evolutionary events, led to 87.1% 16S rRNA gene sequence identity to its closest relative *G. maris*, while the current taxonomic thresholds of the bacterial family border is 86.5%. However, within a family, a minimum sequence identity of 87.65% should be given between the separate species (Yarza et al., 2014), while strain NH11^T^ and *G. maris* comprise only 87.1% sequence identity. Thus without taking evolutionary events into account, strain NH11^T^ falls right in between both thresholds (Yarza et al., 2014). To solve this conflict, a 16S rRNA sequence alignment based cluster analysis was performed. The results (Figures S1–S3) clearly demonstrate that all available 16S rRNA gene sequences from cultivated and uncultivated members of the family *Planctomycetaceae* form multiple clusters, while strain NH11^T^ and the genera *Gimesia* and *Rubinisphaera* belong to three distinct clusters. Thus from a phylogenetic perspective strain NH11^T^ belongs to a novel family.

This conclusion is consistent with other types of evidence obtained in this study: the morphological features of strain NH11^T^ differ from those of its closest relatives. For example, *G. maris* is described to stain Gram negative (Bauld and Staley, 1976) whereas employing the same Gram staining method to strain NH11^T^ delivered no clear result. However, others and we recently demonstrated the presence of peptidoglycan in a phylogenetically representative set of planctomycetal model species (Jeske et al., 2015; van Teeseling et al., 2015). Consequently, we suggest to exclude this classical Gram staining test as a valid tool for future characterization of novel planctomycetal strains, as it is of limited explanatory power for Planctomycetes. Nevertheless, other aspects of strain NH11^T^ differ from its peers as well. Another aspect that differs between strain NH11^T^ and members of the family *Planctomycetaceae* such as *P. limnophila* is the localization of the crateriform structures. They are hardly visible (Figure 3) and seem to be associated with fiber-like structures. However, such fiber-like structures might be artifacts from EM fixation methods and thus require further investigation. If such structures are associated with cell attachment to surfaces remains as well enigmatic. While *G. maris* and *R. brasiliensis* form stalks and rosettes in culture, strain NH11^T^ lacks both features. In addition, strain NH11^T^ forms a tubular connection between daughter and mother cell (Figures 4D–F). Within this tubes in some sections a dark structure became visible that was previously identified as cell division ring in anammox Planctomycetes (van Niftrik et al., 2009). However, anammox bacteria divide, in contrast to budding members of the order Planctomycteales, through binary fission. While both types of organisms lack the otherwise universal cell division protein FtsZ, only for anammox Planctomycetes a potential substitute is known (locus tag kustd1438, NCBI Accession Number CAJ72183). While no homolog protein could be determined in the genome of strain NH11^T^, the molecular mechanism of its unusual cell division remains enigmatic. One might speculate that Fuma_00096, a protein similar, yet distinct from kustd1438, might be involved in the formation of the unique tubular connection between mother and daughter cell and might correlate with the anecdotally observed structure (Figure 4D, black arrow). This idea is based on the Lamin Tail domain of Fuma_00096, which corresponds to an intermediate filament (IF). Such IFs are frequently found in eukaryotic cells to provide mechanical strength and support for fragile tubulin structures (Goldman et al., 1996). However, this is only a hypothesis at this stage, that requires experimental verification.

Besides morphological differences, the genome of strain NH11^T^ shows distinct features if compared to other Planctomycetes as well. First, it contains areas of unique genes, if compared against the available high quality planctomycetal genomes. Second, these areas were found to comprise both, genomic islands - likely acquired through horizontal gene transfer - and giant genes. In total, 19 giant genes with a size >10 kb were detected, which is the highest number amongst all analyzed planctomycetal genomes (Figure S7). Representatives of the family *Planctomycetaceae* in contrast possess a maximum of 13 giant genes of this dimension. The protein products of giant genes, if synthetized, would represent a huge metabolic burden, suggesting that they have an important cellular function. Thus, 6–19 additional giant genes >10 kb compared to its peers suggests a huge difference in strain NH11^T^'s cell surface or secondary metabolism. This is because more than 90% of giant genes encode either surface proteins or polyketide/nonribosomal peptide synthetases (PKS/NRPS) (Reva and Tümmler, 2008). Surprisingly, our antiSMASH analysis revealed only four secondary metabolite-associated gene clusters. One of these gene clusters is predicted to encode the compatible solute ectoine, giving a hint toward an increased salt tolerance of the strain. However, since ASW is tolerated up to 230% (v/v) and NaCl up to 5% (w/v), it is rather adapted to moderate salt concentration. In contrast to the four clusters found in strain NH11^T^'s genome, its closest relatives *R. brasiliensis* and *G. maris* comprise 8 and 9 of such clusters (Jeske et al., 2013). Furthermore, a significant linear correlation between genome size and number of secondary metabolite related genes has been described (Jeske et al., 2013). With a genome size of almost 9 MB strain NH11^T^ is an exception to this observation. Two potential scenarios could be envisioned: either the giant genes of strain NH11^T^ fulfill other, maybe structural functions, or they might be involved in the formation of yet unknown secondary metabolites. While the latter hypothesis fits to the recent observation of novel antibiotic small molecules from Planctomycetes (Jeske et al., 2016), at least some giant genes are predicted to encode proteins with structural function such as the discussed Fuma_00096. While resolving this issue is beyond the scope of this study, we conclude that the genome of strain NH11^T^ differs in important aspects from its peers.

Taken together, the 16S rRNA gene sequence identity, the unusual mode of cell division, its unique cell plan and its unusual planctomycetal genome require to place this novel and exceptional species in a new family. However, the family Planctomycetaceae would not be monophyletic anymore and based on our cluster analysis requires division into three distinct families. To prevent back- and forth renaming of species, genera and families, we describe strain NH11^T^ as novel genus and species for now. We soon will present multiple novel planctomycetal strains and only in the light of such isolates rewriting the planctomycetal taxonomy would make sense. Thus, rearrangements within the family *Planctomycetaceae* will be revisited in the future (Jogler C, personal communication).

### Description of *Fuerstia* gen. nov.

*Fuerstia* (named in honor of John Fuerst, an Australian microbiologist from University of Queensland, who played a key role in planctomycetal research). The pear to ovoid shaped cells form aggregates in liquid culture, but no rosettes. Daughter cells are motile, while mother cells are non-motile and no stalk formation was observed. The surface is smooth, crateriform stuctures are limited to one pole and cells reproduce by polar budding while mother- and daughter cells are connected by a thin tubular-like structure. The lifestyle is heterotrophic, obligatory aerobic and mesophilic. The major fatty acids are C_16:1_ ω6c/_16:1_ ω7c (Summed feature), C_18:1_ω6c/_18:1_ ω7c (Summed feature), and C_16:0_. Member of the phylum Planctomycetes, class Planctomycae, order Planctomycetales, family *Planctomycetaceae*. The type species is *Fuerstia marisgermanicae*.

### Description of *Fuerstia marisgermanicae* sp. nov.

*Fuerstia marisgermanicae* (ma′ris L. n. maris of the sea and ger.ma'ni.cae L. adj. German, pertaining to the German North Sea from which the type strain was isolated). In addition to the features described for the genus, the species exhibits the following properties. Colonies on solid medium are cream colored. Cells are 1.2–2.5 × 0.9–1.7 μm in size. Non motile mother cells spawn motile, swimming, daughter cells. Gram staining delivers no clear result. KOH test and aminopeptidase test are negative, while oxidase and catalase tests positive. The organism is able to degrade a wide range of carbon sources, in particular N-acetyl-D-galactosamine, N-acetyl-D-glucosamine, L-arabinose, D-cellobiose, D-galactose, gentiobiose, α-D-glucose, α-D-lactose, lactulose, maltose, D-mannose, D-melibiose, β-methyl-D-glucoside, sucrose, D-trehalose, turanose, succinic acid mono-methyl-ester, acetic acid, γ-hydroxybutyric acid, itaconic acid, propionic acid, and glycerol were utilized. The enzymatic repertoire of the species, tested with API ZYM, is listed in Table 3. Growth occurs between pH 6 and 10 with an optimum at pH 7. At least 27.5% ASW is needed for growth, The optimal temperature for growth is 28°C (range between 20 and 30°C). The type strain is NH11^T^ (= DSM 27554 = LMG 27831) isolated from a crustacean shell.

## Author contributions

TK analyzed the data and wrote the manuscript. AH performed the physiological experiments and proofread the manuscript. MJ was involved in planning the project, aided in cultivation and helped writing and proofread the manuscript. JV, AK developed the pipeline for the genome analysis. CB prepared thin sections of the strain and performed TEM imaging, was involved in writing and proofreading the manuscript. JO, BB did the genome sequencing of the strain and were involved writing and proofreading the manuscript. PR accomplished phylogenetic analyses, helped writing and proofreading the manuscript. DB performed light-microscopic observations. IG performed the initial cultivation and isolated the stain. HF analyzed the genome for giant genes and proofread the manuscript. HK conducted the fatty acid analysis and proofread the manuscript. MR did the scanning electron microscopy of the strain, TEM imaging of negatively stained cells and proofread the manuscript. SW was involved in the genome analysis and did the multilocus sequence analysis and proofread the manuscript. CJ designed the project and wrote the manuscript.

### Conflict of interest statement

The authors declare that the research was conducted in the absence of any commercial or financial relationships that could be construed as a potential conflict of interest.

## Supplementary material

Figure S1 **Maximum likelihood tree of the detected clusters of NH11T, *Gimesia maris*, and *Rubinispharea brasiliensis* using a minimum sequence identity threshold of 87.65% within a cluster corresponding to phylogenetic families**. All three cultivated species form distinct clusters with sequences of uncultivated planctomycetes with at least 87.65% similarity within their cluster. The corresponding clusters are marked with brackets. The bootstrap percentages of 1000 resamplings are given for each node. *Anammox planctomycetes* served as outgroup.

Figure S2 **Maximum parsimony tree of the detected clusters of NH11T, *Gimesia maris*, and *Rubinispharea brasiliensis* using a minimum sequence identity threshold of 87.65% within a cluster corresponding to phylogenetic families**. All three cultivated species form distinct clusters with sequences of uncultivated planctomycetes with at least 87.65% similarity within their cluster. The corresponding clusters are marked with brackets. The bootstrap percentages of 1000 resamplings are given for each node. *Anammox planctomycetes* served as outgroup.

Figure S3 **Neighbor joining tree of the detected clusters of NH11T, *Gimesia maris*, and *Rubinispharea brasiliensis* using a minimum sequence identity threshold of 87.65% within a cluster corresponding to phylogenetic families**. All three cultivated species form distinct clusters with sequences of uncultivated planctomycetes with at least 87.65% similarity within their cluster. The corresponding clusters are marked with brackets. The bootstrap percentages of 1000 resamplings are given for each node. *Anammox planctomycetes* served as outgroup.

Figure S4 **Temperature, pH, ASW, and NaCl optimum of strain NH11T**. To determine the pH **(A)**, NaCl **(B)**, and ASW **(C)** tolerance as well as the optimal growth temperature **(D)**, the optical density was measured at 600 nm (OD 600 nm) and slope values, corresponding to change of OD 600 nm over time during exponential growth phase, were plotted against the corresponding pH, NaCl, ASW, or temperature value. Growth is best at pH 7, NaCl concentrations up to 5% are tolerated and ASW is tolerated from 27.5 to 230%. Growth is best at 28°C. Each dot represents the mean of triplicate measurements.

Figure S5 **Number of giant genes >5 kb amongst the analyzed strains ordered by declining phylogenetic relationship**. Strain NH11T comprises 45 giant genes >5 kb, which is the second highest number after *Zavarzinella Formosa* with 60 giant genes above this size.

Figure S6 **Number of giant genes >20 kb amongst the analyzed strains ordered by declining phylogenetic relationship**. Strain NH11T comprises five giant genes >20 kb, which is the second highest number after *Rhodopirellula* sp. K833 with seven giant genes above this threshold.

Figure S7 **Number of giant genes >10 kb amongst the analyzed strains ordered by declining phylogenetic relationship**. With 19 giant genes >10 kb, Strain NH11T comprises the highest number above this threshold.
