# Corrigendum: *Fuerstia marisgermanicae* gen. nov., sp. nov., an Unusual Member of the Phylum Planctomycetes From the German Wadden Sea

**DOI:** 10.3389/fmicb.2019.01029

**Published:** 2019-05-15

**Authors:** Timo Kohn, Anja Heuer, Mareike Jogler, John Vollmers, Christian Boedeker, Boyke Bunk, Patrick Rast, Daniela Borchert, Ines Glöckner, Heike M. Freese, Hans-Peter Klenk, Jörg Overmann, Anne-Kristin Kaster, Manfred Rohde, Sandra Wiegand, Christian Jogler

**Affiliations:** ^1^Leibniz Institut Deutsche Sammlung Von Mikroorganismen und Zellkulturen, Braunschweig, Germany; ^2^School of Biology, Newcastle University, Newcastle, United Kingdom; ^3^Helmholtz Centre for Infectious Disease, Braunschweig, Germany

**Keywords:** planctomycetes, *Fuerstia marisgermanicae*, giant genes, cell division, animal associated

In the original article, there was an error. In the Discussion of the article, we described the novel genus “*Fuerstia*,” named to honor John Fuerst, and “*Fuerstia marisgermanicae*,” the proposed type species of the genus. These names were effectively published in Frontiers in Microbiology, but they cannot be validly published based on the Rules of the International Code of Nomenclature of Prokaryotes. The proposed generic name cannot be used as a generic name for a new prokaryote as it is illegitimate based on Principle 2 of the Code. Moreover, the specific epithet is malformed. Therefore, we present corrected names that are here effectively published and will be submitted for validation according to the Rules of the Code.

A correction has been made to the **Discussion**, subsection **Description of** ***Fuerstia gen*.** ***nov*.***:*

“*Fuerstiella* (Fuer.sti.el'la. N.L. dim. fem. n. *Fuerstiella*, named in honor of John Fuerst, an Australian microbiologist from University of Queensland, who played a key role in planctomycetal research). The pear to ovoid shaped cells form aggregates in liquid culture, but no rosettes. Daughter cells are motile, while mother cells are non-motile and no stalk formation was observed. The surface is smooth, crateriform structures are limited to one pole and cells reproduce by polar budding while mother- and daughter cells are connected by a thin tubular-like structure. The lifestyle is heterotrophic, obligatory aerobic and mesophilic. The major fatty acids are C_16:1_ ω6c/_16:1_ ω7c (Summed feature), C_18:1_ ω6c/_18:1_ ω7c (Summed feature), and C_16:0_. Member of the phylum Planctomycetes, class Planctomycae, order Planctomycetales, family *Planctomycetaceae*. The type species is *Fuerstiella marisgermanici*.”

Additionally, a correction has been made to the **Description**, subsection ***Fuerstia*** ***marisgermanicae* sp. nov**.:

“*Fuerstiella marisgermanici* (ma.ris.ger.ma'ni.ci. L. neut. n. mare the sea; L. masc. adj. *germanicus* German; N.L. gen. n. *marisgermanici* of the German sea, pertaining to the German North Sea from which the type strain was isolated). In addition to the features described for the genus, the species exhibits the following properties. Colonies on solid medium are cream colored. Cells are 1.2–2.5 × 0.9–1.7 μm in size. Non-motile mother cells spawn motile, swimming, daughter cells. Gram staining delivers no clear result. KOH test and aminopeptidase test are negative, while oxidase and catalase tests positive. The organism is able to degrade a wide range of carbon sources, in particular N-acetyl-D-galactosamine, N-acetyl-D-glucosamine, L-arabinose, D-cellobiose, D-galactose, gentiobiose, α-D-glucose, α-D-lactose, lactulose, maltose, D-mannose, D-melibiose, β-methyl-D-glucoside, sucrose, D-trehalose, turanose, succinic acid mono-methyl-ester, acetic acid, γ-hydroxybutyric acid, itaconic acid, propionic acid, and glycerol were utilized. The enzymatic repertoire of the species, tested with API ZYM, is listed in Table 3 of the original publication. Growth occurs between pH 6 and 10 with an optimum at pH 7. At least 27.5% ASW is needed for growth, the optimal temperature for growth is 28°C (range between 20 and 30°C). The type strain is NH11^T^ (= DSM 27554 = LMG 27831) isolated from a crustacean shell.”

The authors apologize for this error and state that this does not change the scientific conclusions of the article in any way.

