# Supplementary figures and images for: Fuerstia marisgermanicae gen. nov., sp. nov., an Unusual Member of the Phylum Planctomycetes from the German Wadden Sea
Source: Front Microbiol. 2016 Dec 22;7:2079. doi: 10.3389/fmicb.2016.02079 (PMC5177795; doi:10.3389/fmicb.2016.02079)

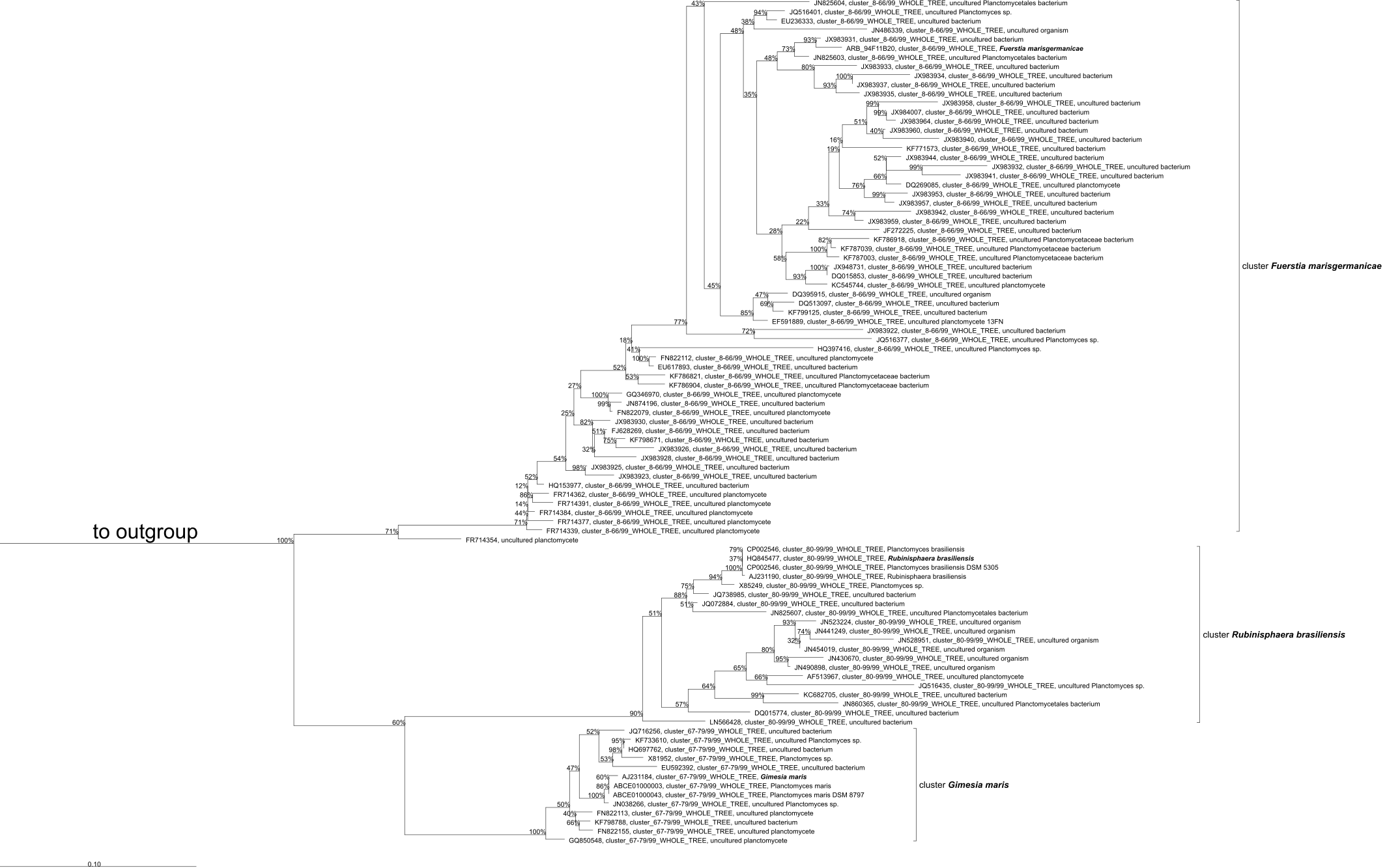

Supplement: Figure S1 — Maximum likelihood tree of the detected clusters of NH11T, Gimesia maris, and Rubinispharea brasiliensis using a minimum sequence identity threshold of 87.65% within a cluster corresponding to phylogenetic families. All three cultivated species form distinct clusters with sequences of uncultivated planctomycetes with at least 87.65% similarity within their cluster. The corresponding clusters are marked with brackets. The bootstrap percentages of 1000 resamplings are given for each node. Anammox planctomycetes served as outgroup. [file Image1.TIF]

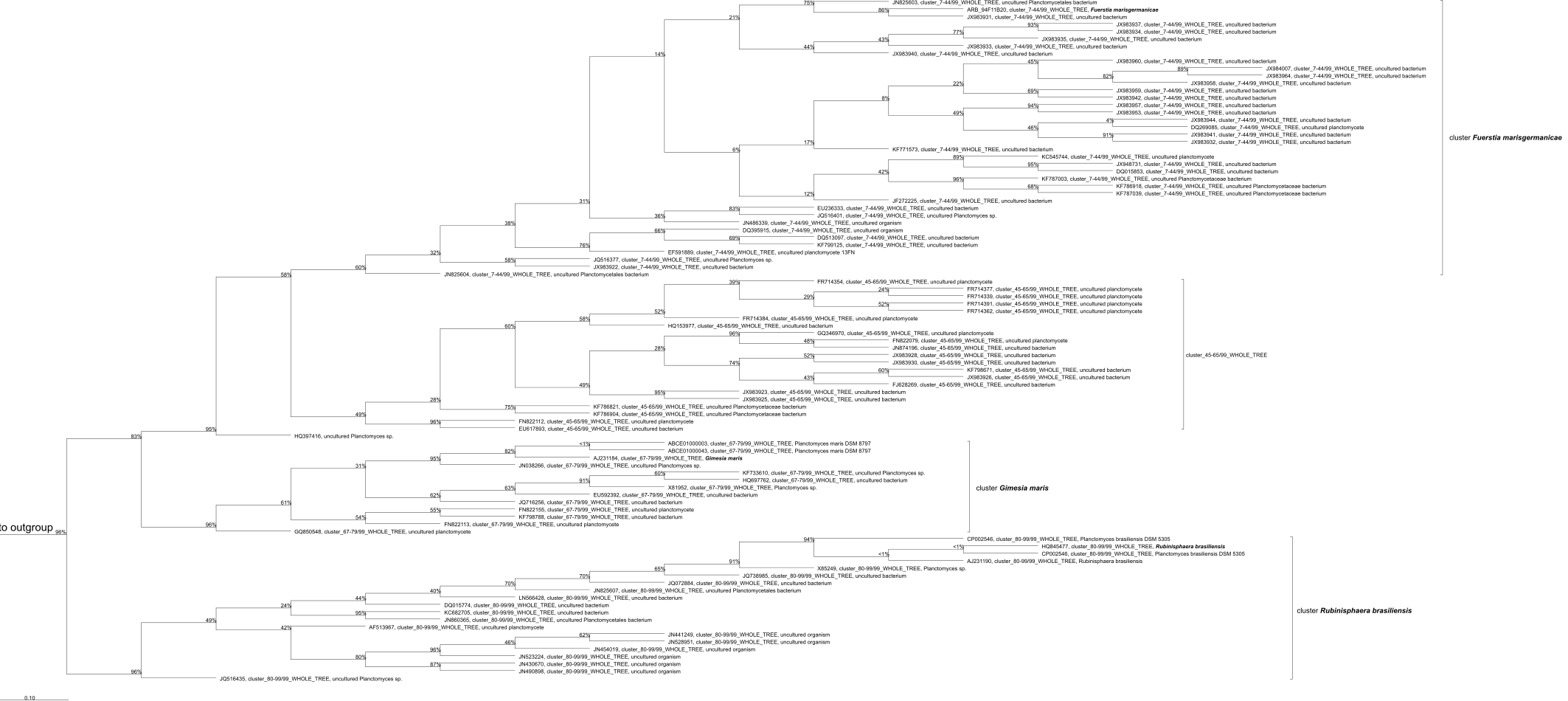

Supplement: Figure S2 — Maximum parsimony tree of the detected clusters of NH11T, Gimesia maris, and Rubinispharea brasiliensis using a minimum sequence identity threshold of 87.65% within a cluster corresponding to phylogenetic families. All three cultivated species form distinct clusters with sequences of uncultivated planctomycetes with at least 87.65% similarity within their cluster. The corresponding clusters are marked with brackets. The bootstrap percentages of 1000 resamplings are given for each node. Anammox planctomycetes served as outgroup. [file Image2.TIF]

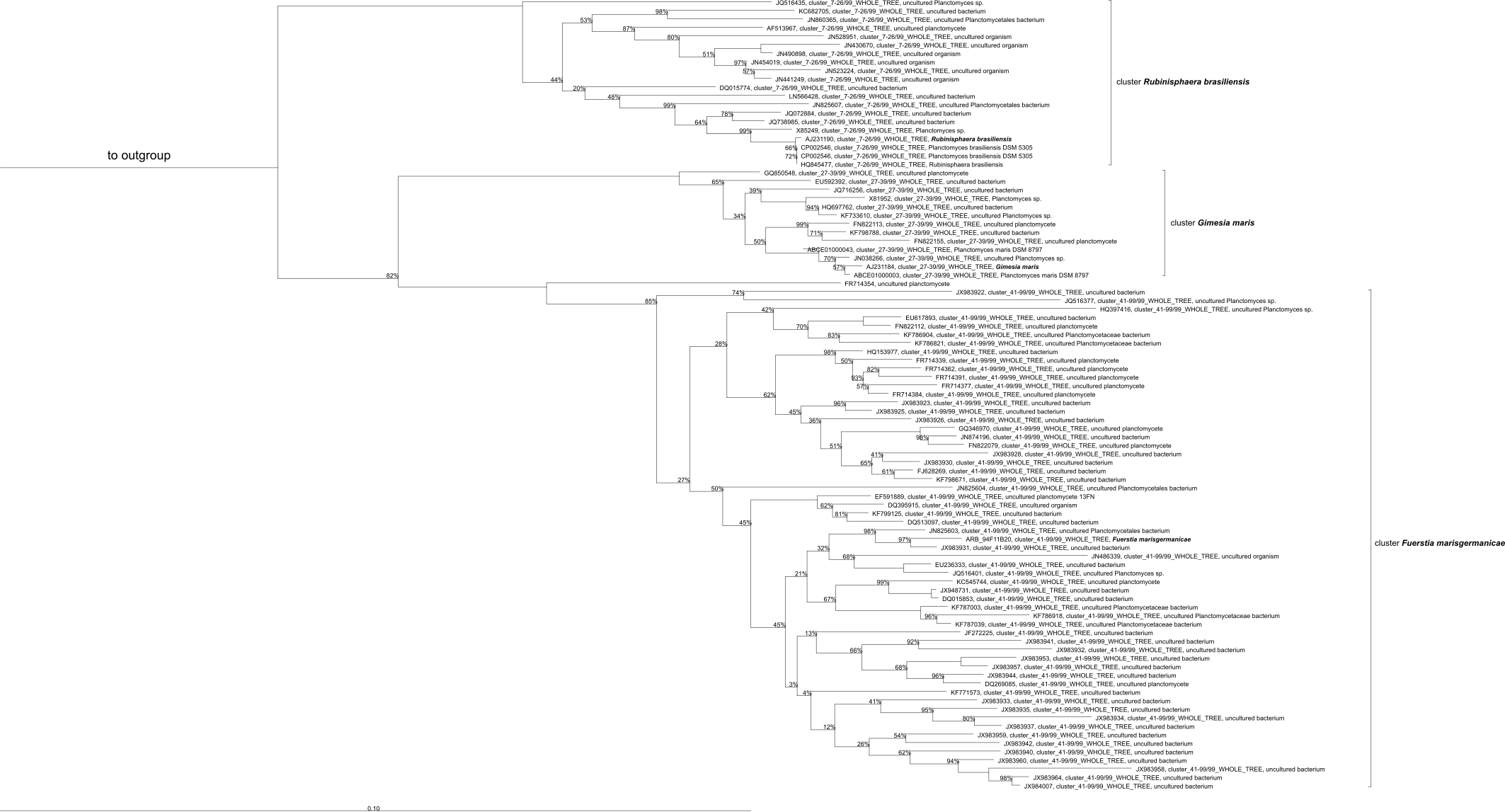

Supplement: Figure S3 — Neighbor joining tree of the detected clusters of NH11T, Gimesia maris, and Rubinispharea brasiliensis using a minimum sequence identity threshold of 87.65% within a cluster corresponding to phylogenetic families. All three cultivated species form distinct clusters with sequences of uncultivated planctomycetes with at least 87.65% similarity within their cluster. The corresponding clusters are marked with brackets. The bootstrap percentages of 1000 resamplings are given for each node. Anammox planctomycetes served as outgroup. [file Image3.TIF]

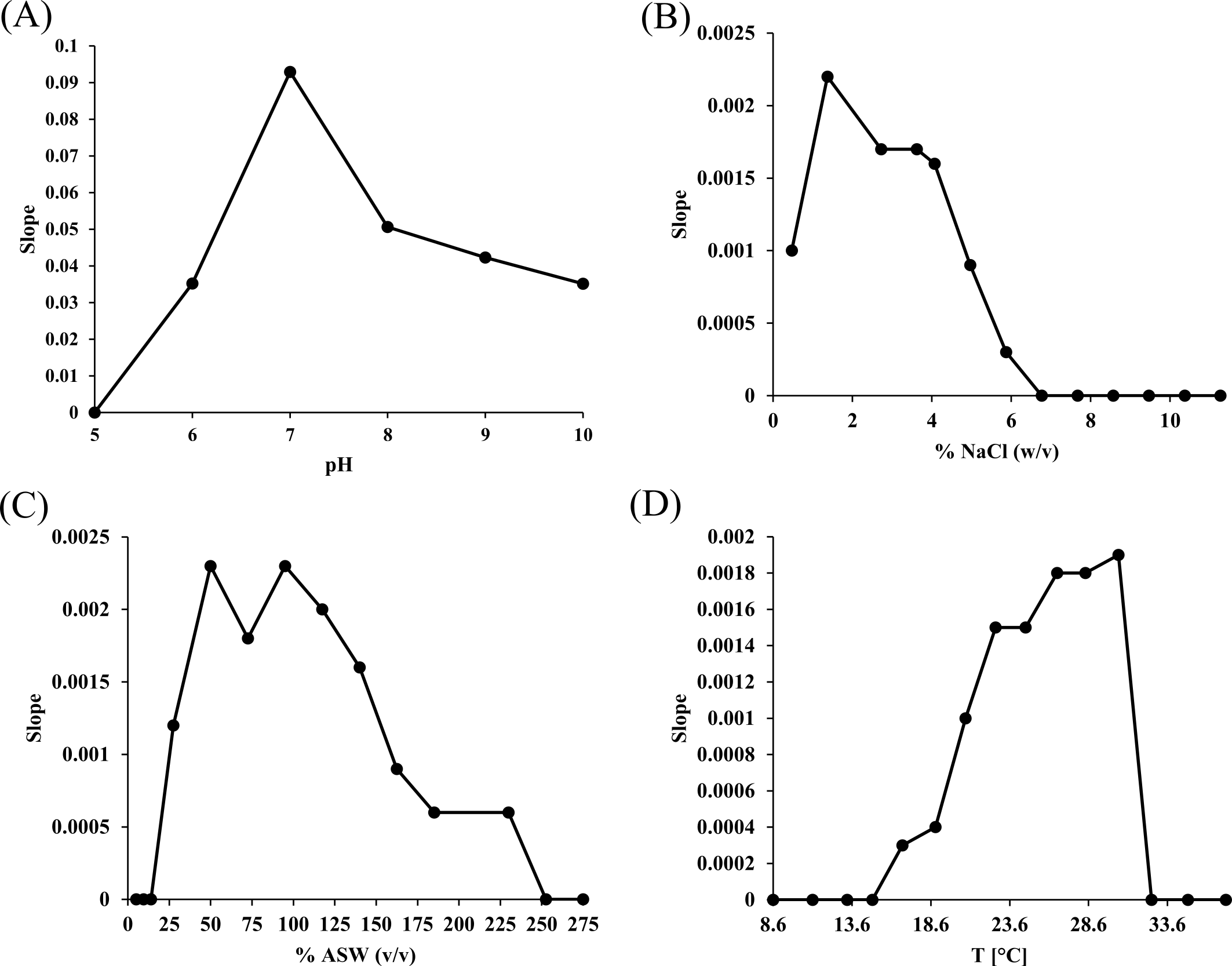

Supplement: Figure S4 — Temperature, pH, ASW, and NaCl optimum of strain NH11T. To determine the pH (A), NaCl (B), and ASW (C) tolerance as well as the optimal growth temperature (D), the optical density was measured at 600 nm (OD 600 nm) and slope values, corresponding to change of OD 600 nm over time during exponential growth phase, were plotted against the corresponding pH, NaCl, ASW, or temperature value. Growth is best at pH 7, NaCl concentrations up to 5% are tolerated and ASW is tolerated from 27.5 to 230%. Growth is best at 28°C. Each dot represents the mean of triplicate measurements. [file Image4.TIF]

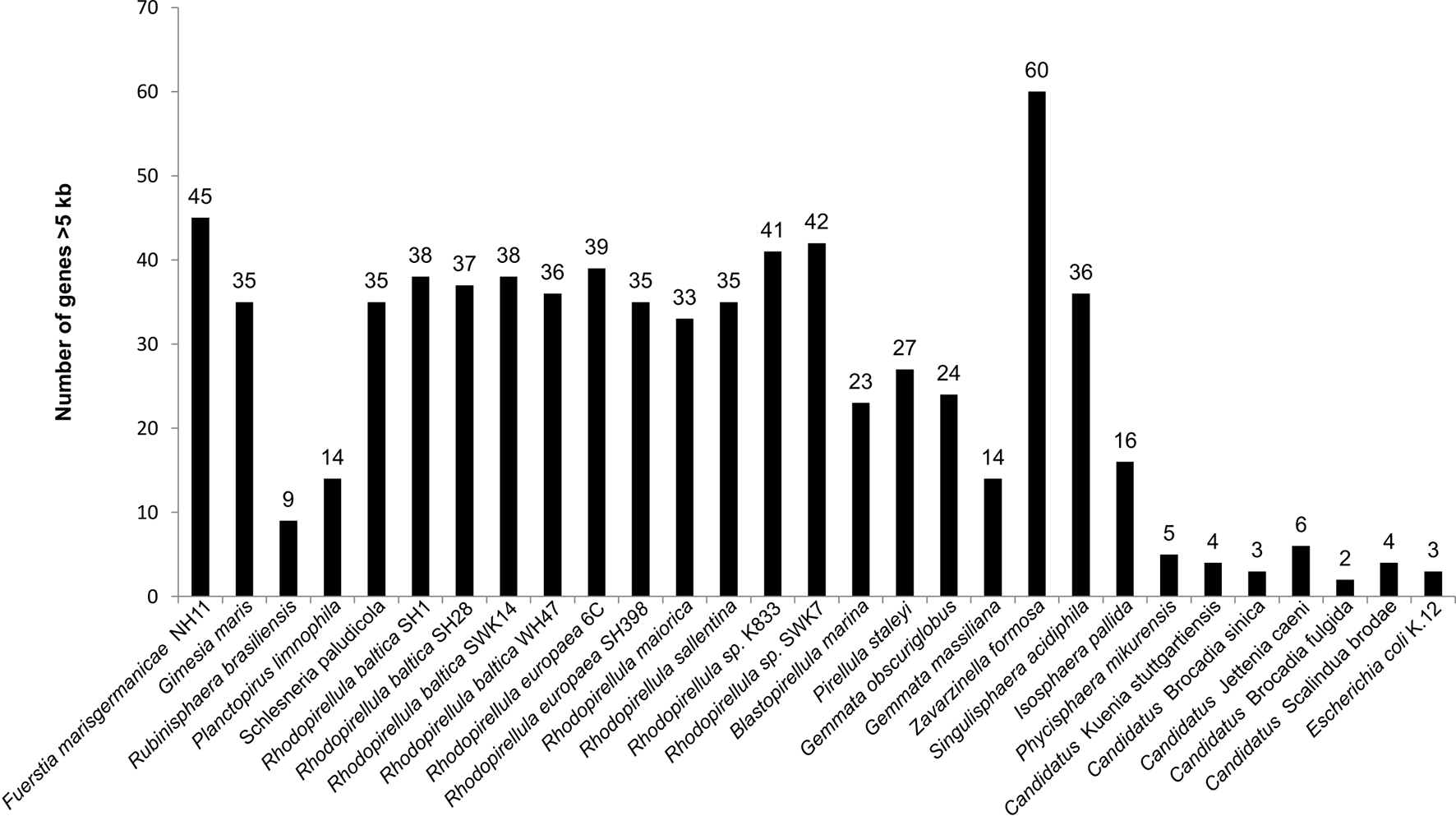

Supplement: Figure S5 — Number of giant genes >5 kb amongst the analyzed strains ordered by declining phylogenetic relationship. Strain NH11T comprises 45 giant genes >5 kb, which is the second highest number after Zavarzinella Formosa with 60 giant genes above this size. [file Image5.TIF]

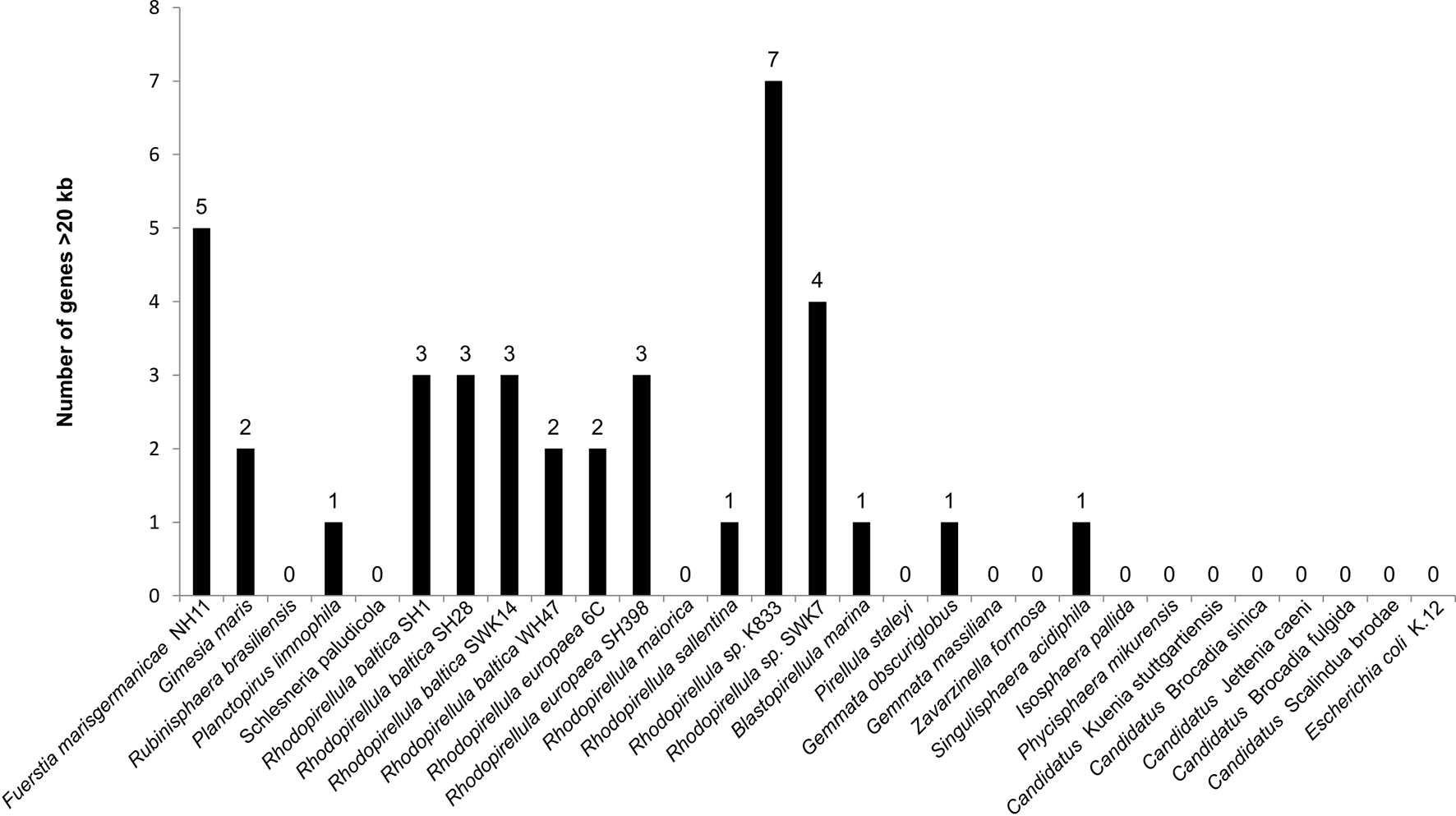

Supplement: Figure S6 — Number of giant genes >20 kb amongst the analyzed strains ordered by declining phylogenetic relationship. Strain NH11T comprises five giant genes >20 kb, which is the second highest number after Rhodopirellula sp. K833 with seven giant genes above this threshold. [file Image6.TIF]

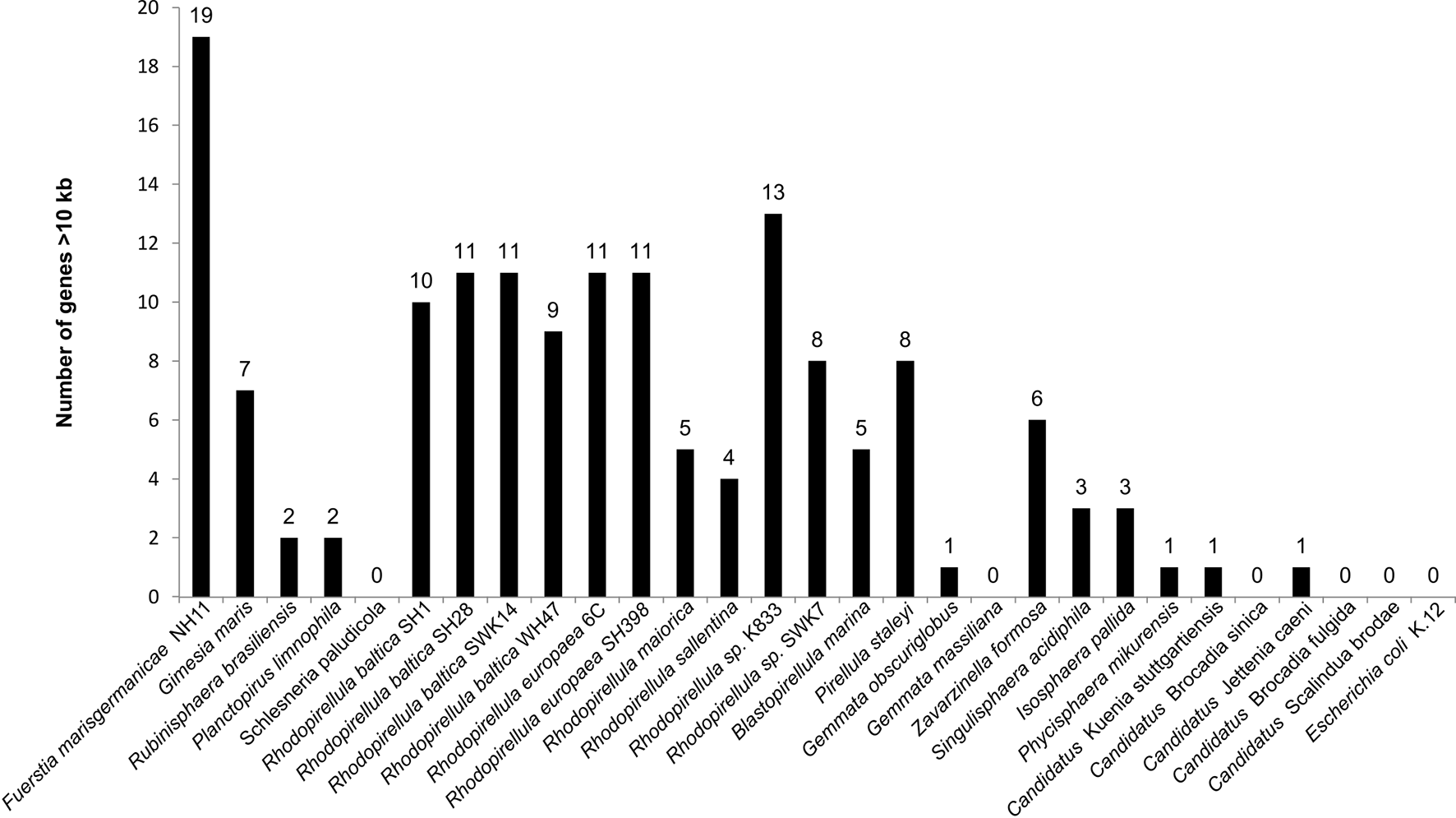

Supplement: Figure S7 — Number of giant genes >10 kb amongst the analyzed strains ordered by declining phylogenetic relationship. With 19 giant genes >10 kb, Strain NH11T comprises the highest number above this threshold. [file Image7.TIF]
