# Supplementary material for: Fuerstia marisgermanicae gen. nov., sp. nov., an Unusual Member of the Phylum Planctomycetes from the German Wadden Sea
Source: Front Microbiol. 2016 Dec 22;7:2079. doi: 10.3389/fmicb.2016.02079 (PMC5177795; doi:10.3389/fmicb.2016.02079)
Supplement: Supplementary file 8 [file DataSheet1.DOCX]

**Figure S1 | Maximum likelihood tree of the detected clusters of NH11^T^, *Gimesia maris* and *Rubinispharea brasiliensis* using a minimum sequence identity threshold of 87.65% within a cluster corresponding to phylogenetic families*.*** All three cultivated species form distinct clusters with sequences of uncultivated planctomycetes with at least 87.65% similarity within their cluster. The corresponding clusters are marked with brackets. The bootstrap percentages of 1000 resamplings are given for each node. Anammox planctomycetes served as outgroup.

**Figure S2 | Maximum parsimony tree of the detected clusters of NH11^T^, *Gimesia maris* and *Rubinispharea brasiliensis* using a minimum sequence identity threshold of 87.65% within a cluster corresponding to phylogenetic families.** All three cultivated species form distinct clusters with sequences of uncultivated planctomycetes with at least 87.65% similarity within their cluster. The corresponding clusters are marked with brackets. The bootstrap percentages of 1000 resamplings are given for each node. Anammox planctomycetes served as outgroup.

**Figure S3 | Neighbor joining tree of the detected clusters of NH11^T^, *Gimesia maris* and *Rubinispharea brasiliensis* using a minimum sequence identity threshold of 87.65% within a cluster corresponding to phylogenetic families.** All three cultivated species form distinct clusters with sequences of uncultivated planctomycetes with at least 87.65% similarity within their cluster. The corresponding clusters are marked with brackets. The bootstrap percentages of 1000 resamplings are given for each node. Anammox planctomycetes served as outgroup.

**Figure S4 | Temperature, pH, ASW and NaCl optimum of strain NH11**^T^**.** To determine the pH **(A)**, NaCl **(B)** and ASW **(C)** tolerance as well as the optimal growth temperature **(D)**, the optical density was measured at 600 nm (OD_600nm_) and slope values, corresponding to change of OD_600nm_ over time during exponential growth phase, were plotted against the corresponding pH, NaCl, ASW or temperature value. Growth is best at pH 7, NaCl concentrations up to 5 % are tolerated and ASW is tolerated from 27.5 % - 230 %. Growth is best at 28 °C.. Each dot represents the mean of triplicate measurements.

**Figure S5 | Number of giant genes >5kb amongst the analyzed strains ordered by declining phylogenetic relationship.** Strain NH11^T^ comprises 45 giant genes >5kb, which is the second highest number after *Zavarzinella Formosa* with 60 giant genes above this size.

**Figure S6 | Number of giant genes >20kb amongst the analyzed strains ordered by declining phylogenetic relationship.** Strain NH11^T^ comprises 5 giant genes >20kb, which is the second highest number after *Rhodopirellula* sp. K833 with 7 giant genes above this threshold.

**Figure S7 | Number of giant genes >10kb amongst the analyzed strains ordered by declining phylogenetic relationship.** With 19 giant genes >10kb, Strain NH11^T^ comprises the highest number above this threshold.
