# Supplementary material for: Fuerstia marisgermanicae gen. nov., sp. nov., an Unusual Member of the Phylum Planctomycetes from the German Wadden Sea
Source: Front Microbiol. 2016 Dec 22;7:2079. doi: 10.3389/fmicb.2016.02079 (PMC5177795; doi:10.3389/fmicb.2016.02079)
Supplement: Supplementary file 9 [file Table1.DOCX]

**Table S1 | List of reference and out-group strains, with the corresponding accession numbers, used for phylogenetic tree reconstruction and giant gene analysis.**

| **Species** | **Strain** | | **Genome accession number** | | **16S rRNA gene accession number** | |
| --- | --- | --- | --- | --- | --- | --- |
| *Algisphaera agarilytica* | 06SJR6-2 | | - | | AB845176 | |
| *Aquisphaera giovannonii* | OJF2^T^ | | - | | NR_122081 | |
| *Bythopirellula goksoyri* | Pr1d | | - | | NR_118636 | |
| *Blastopirellula cremea* | LHWP2^T^ | | - | | NR_118153 | |
| *Blastopirellula marina* | SH 106^T^ | | NZ_AANZ00000000 | | NR_029226 | |
| *Ca.* Gemmata massiliana | IIL30 | | CBXA000000000 | | JX088244 | |
| *Gemmata obscuriglobus* | UQM 2246^T^ | | NZ_ABGO00000000 | | NR_114712 | |
| *Gimesia maris* | 534-30^T^ | | NZ_ABCE00000000 | | NR_025327 | |
| *Isosphaera pallida* | IS1B^T^ | | NC_014962.1 | | NR_074534 | |
| *Paludisphaera borealis* | PX4^T^ | | - | | KT372165 | |
| *Phycisphaera mikurensis* | FYK2301M01^T^ | | NC_017080.1 | | NR_074491 | |
| Phycisphaera bacterium bin0 | - | | 2593339136 (IMG ID) | | Gp0110150 (GOLD ID) | |
| *Pirellula staleyi* | ATCC 27377^T^ | | NC_013720.1 | | NR_074521 | |
| *Planctomyces sp.* SH-PL62 | - | | CP011273.1 | | - | |
| *Planctomicrobium piriforme* | P3 | | - | | KP161655 | |
| *Planctopirus limnophila* | Mü 290^T^ | | NC_014148.1 | | NR_074670 | |
| *Rhodopirellula baltica* | SH1^T^ | | NC_005027.1 | | NR_043384 | |
| *Rhodopirellula baltica* | SH28 | | AMCW01000001 | | - | |
| *Rhodopirellula baltica* | SWK14 | | AMWG01000001 | | - | |
| *Rhodopirellula baltica* | WH47 | | AFAR01000001 | | - | |
| *Rhodopirellula europaea* | 6C | | ANMO01000001 | | - | |
| *Rhodopirellula europaea* | SH398 | | ANOF01000001 | | - | |
| *Rhodopirellula lusitana* | UC17 | | - | | EF589351 | |
| *Rhodopirellula maiorica* | SM1 | | ANOG00000000 | | FJ624363 | |
| *Rhodopirellula rosea* | LHWP3 | | - | | JF748734 | |
| *Rhodopirellula rubra* | SWK7 | | ANOQ00000000 | | FJ624377 | |
| *Rhodopirellula rubra* | LF2^T^ | | - | | HQ845500 | |
| *Rhodopirellula sallentina* | SM41 | | ANOH00000000 | | FJ624360 | |
| *Rhodopirellula* sp. | K833 | | LECT01000001 | | - | |
| *Roseimaritima ulvae* | UC8 | | - | | HQ844408 | |
| *Rubinisphaera brasiliensis* | DSM 5305^T^ | | NC_015174.1 | | NR_074297 | |
| *Rubripirellula obstinata* | LF1 | | - | | DQ986201.2 | |
| *Schlesneria paludicola* | MPL7^T^ | | NZ_AHZR00000000 | | NR_042466 | |
| *Singulisphaera acidiphila* | MOB10^T^ | | NC_019892.1 | | NR_102439 | |
| *Singulisphaera rosea* | S26^T^ | | - | | NR_116969 | |
| *Thermopirellula anaerolimosa* | VM20-7 | | - | | AB558583 | |
| *Telmatocola sphagniphila* | SP2^T^ | | - | | NR_118328 | |
| *Tepidisphaera mucosa* | 2842^T^ | | - | | KM036168 | |
| *Zavarzinella formosa* | A10^T^ | | AIAB00000000 | | NR_042465 | |
| *Ca.* Jettenia asiatica | - | | LAQJ01000000 | | DQ301513 | |
| *Ca.* Anammoxoglobus propionicus | - | | - | | EU478694 | |
| *Ca.* Brocadia anammoxidans | - | | - | | AF375994 | |
| *Ca.* Brocadia fulgida | - | | LAQJ01000000 | | DQ459989.1 | |
| *Ca.* Kuenenia stuttgartiensis | - | | CT030148 | | CT573071 | |
| *Ca.* Scalindua wagneri | - | | - | | EU478692 | |
| *Ca.* Jettenia caeni | - | | BAFH01000000 | | AB057453.1 | |
| *Ca.* Scalindua brodae | - | | JRYO01000000 | | AY257181 | |
| *Ca.* Brocadia sinica | JPN1 | | BAFN01000000 | | AB565477 | |
| *Escherichia coli* | K12 | | NC_000913 | | - | |
| Outgroup sequences | | | | | | |
| *Brevifollis gellanilyticus* | | DC2c-G4 | | - | | AB552872 |
| *Verrucomicrobium spinosum* | | DSM 4136 | | - | | NR_026266 |
| *Chthoniobacter flavus* | | Ellin428 | | - | | NR_115225 |
| *Akkermansia muciniphila* | | Muc | | - | | AY271254 |
| *Limisphaera ngatamarikiensis* | | NGM72.4^T^ | | - | | HF947551 |
| *Alterococcus agarolyticus* | | ADT3 | | - | | AF075271 |
| *Opitutus terrae* | | PB90-1 | | - | | AJ229235 |
