# Supplementary material for: Fuerstia marisgermanicae gen. nov., sp. nov., an Unusual Member of the Phylum Planctomycetes from the German Wadden Sea
Source: Front Microbiol. 2016 Dec 22;7:2079. doi: 10.3389/fmicb.2016.02079 (PMC5177795; doi:10.3389/fmicb.2016.02079)
Supplement: Supplementary file 10 [file Table2.DOCX]

Table S2 | Differences in 16S rRNA gene sequence among members of the phylum Planctomycetes

Pairwise distances of the strains based on the 1,270 base pairs analyzed in phylogenetic reconstruction. Numbers represent the percentage of identity with the felsenstein correction implemented. Values of strain NH11^T^ are shaded in grey.

|  | *Rhodopirellula* group | *Rubripirellula obstinata* | *Roseimaritima ulvae* | *Blastopirellula cremea* | *Blastopirellula marina* | *Pirellula staleyi* | *Bythopirellula goksoyri* | Thermophilic Planctomycetes | *Planctomicrobium piriforme* | *Rubinisphaera brasiliensis* | *Fuerstia marisgermanicae* NH11^T^ | *Gimesia maris* | *Planctopirus limnophila* | *Schlesneria paludicola* | *Singulisphaera acidiphila* | *Singulisphaera rosea* | *Aquisphaera giovannonii* | *Paludisphaera borealis* | *Isosphaera pallida* | *Gemmata massiliana* | *Gemmata obscuriglobus* | *Telmatocola sphagniphila* | *Zarvazinella formosa* | *Algisphaera agarilytica* | *Phycisphaera mikurensis* | *Tepidisphaera mucosa* | Anammox Planctomycetes |
| --- | --- | --- | --- | --- | --- | --- | --- | --- | --- | --- | --- | --- | --- | --- | --- | --- | --- | --- | --- | --- | --- | --- | --- | --- | --- | --- | --- |
| *Rhodopirellula* group | 93.8-100 | 92.5-94.7 | 91.9-92.6 | 87-87.6 | 87.2-88.7 | 82.2-83.2 | 82.9-84 | 84.7-84.7 | 78.1-78.7 | 81.3-82.5 | 79.4-80.7 | 82.7-83.3 | 78.2-79.2 | 76.3-77.4 | 72.8-74.3 | 74-75.5 | 73.6-75.3 | 74.1-76 | 71.4-72.6 | 72.2-74.6 | 71.7-73.3 | 70.2-71.5 | 72.9-74.9 | 73.1-74.4 | 72.5-74.2 | 70.8-71.7 | 69.5-73.2 |
| *Rubripirellula obstinata* | 92.5-94.7 | 100 | 92.5 | 85.4 | 85.9 | 81.7 | 82.5 | 80-82.5 | 77.8 | 80.7 | 76.8 | 82.3 | 77.4 | 76.7 | 73.7 | 74.8 | 74.5 | 74.5 | 71.7 | 73.2 | 71.3 | 72.1 | 74.3 | 73.7 | 73.7 | 71 | 69.5-71 |
| *Roseimaritima ulvae* | 91.9-92.6 | 92.5 | 100 | 86.3 | 86.6 | 82.8 | 83.2 | 81.2-85 | 78.9 | 81.6 | 78.7 | 82.7 | 79.9 | 77.9 | 74.7 | 76.7 | 76.7 | 77.4 | 73.1 | 75.4 | 74 | 73.7 | 75.5 | 72.4 | 73.2 | 71.7 | 69.2-71.2 |
| *Blastopirellula cremea* | 87-87.6 | 85.4 | 86.3 | 100 | 94.3 | 87.4 | 86.1 | 84.4-84.6 | 79.7 | 83 | 80.1 | 81.5 | 79 | 79.8 | 75 | 75.9 | 76.2 | 75.9 | 73.5 | 77.4 | 75.5 | 74.3 | 76.6 | 72.5 | 71.9 | 71.1 | 71.2-73.6 |
| *Blastopirellula marina* | 87.2-88.7 | 85.9 | 86.6 | 94.3 | 100 | 87 | 86.1 | 84.9-85.4 | 79.7 | 82.6 | 79.2 | 80.8 | 79.5 | 79.7 | 75.7 | 76.6 | 76.1 | 75.6 | 73.5 | 77.2 | 76.3 | 74.2 | 76.6 | 71.5 | 71.8 | 70.6 | 70.5-72.9 |
| *Pirellula staleyi* | 82.2-83.2 | 81.7 | 82.8 | 87.4 | 87 | 100 | 83.7 | 83-83.7 | 78.1 | 78.5 | 78.1 | 81.3 | 78.7 | 77.8 | 74.6 | 74.7 | 75.7 | 75.6 | 73.5 | 77.1 | 74.9 | 75.3 | 75.7 | 71.6 | 72 | 73.7 | 69-71.1 |
| *Bythopirellula goksoyri* | 82.9-84 | 82.5 | 83.2 | 86.1 | 86.1 | 83.7 | 100 | 82.4-85 | 76.2 | 78.4 | 78.6 | 78.8 | 78.4 | 76.5 | 75.4 | 76 | 76.3 | 75.9 | 74.9 | 75.2 | 74.5 | 73 | 74.2 | 70.2 | 73.2 | 74.4 | 68.5-70.6 |
| Thermophilic Planctomycetes | 82.1-84.7 | 80-82.5 | 81.2-85 | 84.4-84.6 | 84.9-85.4 | 83-83.7 | 82.4-85 | 91.8-100 | 80.2-81.2 | 81.4-82.8 | 78.4-79.5 | 80.7-82.4 | 78.3-79.8 | 80.1-80.7 | 75.7-76.6 | 77.2-78.4 | 77.8-78.6 | 77.8-79.1 | 76.6-77 | 77.4-79.4 | 75.7-77.2 | 73.4-75.2 | 75.3-76.2 | 73.5-75.1 | 73.5-75.1 | 72.5-73.7 | 70.2-74.3 |
| *Planctomicrobium piriforme* | 78.1-78.7 | 77.8 | 78.9 | 79.7 | 79.7 | 78.1 | 76.2 | 80.2-81.2 | 100 | 87.1 | 81.6 | 85.4 | 84.3 | 79.7 | 74.3 | 75.8 | 78 | 77.2 | 75.9 | 73.9 | 73.6 | 73 | 75.3 | 71.8 | 72.2 | 68.6 | 67.4-70.5 |
| *Rubinisphaera brasiliensis* | 81.3-82.5 | 80.7 | 81.6 | 83 | 82.6 | 78.5 | 78.4 | 81.4-82.8 | 87.1 | 100 | 84.1 | 87.8 | 82.6 | 80.9 | 75.9 | 77 | 78.8 | 79.2 | 76.6 | 75.4 | 74.7 | 74.4 | 74.6 | 73.2 | 74.3 | 69.6 | 69.2-71.5 |
| *Fuerstia marisgermanicae* NH11^T^ | 79.4-80.7 | 76.8 | 78.7 | 80.1 | 79.2 | 78.1 | 78.6 | 78.4-79.5 | 81.6 | 84.1 | 100 | 85.4 | 81.3 | 79.3 | 71.7 | 72.6 | 73.9 | 75.2 | 72.6 | 72.5 | 71.3 | 72.1 | 71.9 | 70.1 | 70.8 | 70.1 | 67.4-70.4 |
| *Gimesia maris* | 82.7-83.3 | 82.3 | 82.7 | 81.5 | 80.8 | 81.3 | 78.8 | 80.7-82.4 | 85.4 | 87.8 | 85.4 | 100 | 82.9 | 83.3 | 72.8 | 73.6 | 75.2 | 76 | 72.3 | 75.1 | 73.9 | 73.3 | 73.8 | 73.1 | 73.2 | 69.9 | 68-70.1 |
| *Planctopirus limnophila* | 78.2-79.2 | 77.4 | 79.9 | 79 | 79.5 | 78.7 | 78.4 | 78.3-79.8 | 84.3 | 82.6 | 81.3 | 82.9 | 100 | 85.3 | 73.3 | 76.3 | 77.2 | 77.1 | 75.3 | 73.8 | 74.7 | 74.7 | 75.3 | 70.9 | 71.7 | 67.1 | 68.9-70.9 |
| *Schlesneria paludicola* | 76.3-77.4 | 76.7 | 77.9 | 79.8 | 79.7 | 77.8 | 76.5 | 80.1-80.7 | 79.7 | 80.9 | 79.3 | 83.3 | 85.3 | 100 | 72.9 | 74.4 | 75.7 | 75.5 | 71.9 | 73 | 72.2 | 72.2 | 73.5 | 69.6 | 70.1 | 69.1 | 66.6-68.5 |
| *Singulisphaera acidiphila* | 72.8-74.3 | 73.7 | 74.7 | 75 | 75.7 | 74.6 | 75.4 | 75.7-76.6 | 74.3 | 75.9 | 71.7 | 72.8 | 73.3 | 72.9 | 100 | 94.8 | 91.4 | 91.4 | 86 | 73.8 | 74.1 | 73.5 | 74.3 | 67.1 | 68.2 | 69.1 | 67.2-68.3 |
| *Singulisphaera rosea* | 74-75.5 | 74.8 | 76.7 | 75.9 | 76.6 | 74.7 | 76 | 77.2-78.4 | 75.8 | 77 | 72.6 | 73.6 | 76.3 | 74.4 | 94.8 | 100 | 92.1 | 92.4 | 87.6 | 74.3 | 73.5 | 74.1 | 75 | 66.9 | 68.5 | 68.8 | 67.9-68.4 |
| *Aquisphaera giovannonii* | 73.6-75.3 | 74.5 | 76.7 | 76.2 | 76.1 | 75.7 | 76.3 | 77.8-78.6 | 78 | 78.8 | 73.9 | 75.2 | 77.2 | 75.7 | 91.4 | 92.1 | 100 | 94.1 | 89.7 | 74.6 | 74.9 | 73.4 | 75.6 | 68.2 | 70.8 | 69.7 | 68.6-69.3 |
| *Paludisphaera borealis* | 74.1-76 | 74.5 | 77.4 | 75.9 | 75.6 | 75.6 | 75.9 | 77.8-79.1 | 77.2 | 79.2 | 75.2 | 76 | 77.1 | 75.5 | 91.4 | 92.4 | 94.1 | 100 | 90.3 | 75.1 | 74.5 | 73.7 | 74.5 | 68 | 70.2 | 68.1 | 67.9-69.8 |
| *Isosphaera pallida* | 71.4-72.6 | 71.7 | 73.1 | 73.5 | 73.5 | 73.5 | 74.9 | 76.6-77 | 75.9 | 76.6 | 72.6 | 72.3 | 75.3 | 71.9 | 86 | 87.6 | 89.7 | 90.3 | 100 | 73.8 | 73.6 | 73.7 | 74.5 | 67 | 69.4 | 68 | 64.6-68.1 |
| *Gemmata massiliana* | 72.2-74.6 | 73.2 | 75.4 | 77.4 | 77.2 | 77.1 | 75.2 | 77.4-79.4 | 73.9 | 75.4 | 72.5 | 75.1 | 73.8 | 73 | 73.8 | 74.3 | 74.6 | 75.1 | 73.8 | 100 | 94.3 | 82.4 | 84.9 | 69.8 | 70.2 | 72.1 | 66.7-70.3 |
| *Gemmata obscuriglobus* | 71.7-73.3 | 71.3 | 74 | 75.5 | 76.3 | 74.9 | 74.5 | 75.7-77.2 | 73.6 | 74.7 | 71.3 | 73.9 | 74.7 | 72.2 | 74.1 | 73.5 | 74.9 | 74.5 | 73.6 | 94.3 | 100 | 81.2 | 84.3 | 68 | 69.4 | 70.3 | 64.9-68.3 |
| *Telmatocola sphagniphila* | 70.2-71.5 | 72.1 | 73.7 | 74.3 | 74.2 | 75.3 | 73 | 73.4-75.2 | 73 | 74.4 | 72.1 | 73.3 | 74.7 | 72.2 | 73.5 | 74.1 | 73.4 | 73.7 | 73.7 | 82.4 | 81.2 | 100 | 85.6 | 66.8 | 65.6 | 70 | 67-69.7 |
| *Zarvazinella formosa* | 72.9-74.9 | 74.3 | 75.5 | 76.6 | 76.6 | 75.7 | 74.2 | 75.3-76.2 | 75.3 | 74.6 | 71.9 | 73.8 | 75.3 | 73.5 | 74.3 | 75 | 75.6 | 74.5 | 74.5 | 84.9 | 84.3 | 85.6 | 100 | 67.9 | 68 | 72.1 | 67.8-70.9 |
| *Algisphaera agarilytica* | 73.1-74.4 | 73.7 | 72.4 | 72.5 | 71.5 | 71.6 | 70.2 | 73.5-75.1 | 71.8 | 73.2 | 70.1 | 73.1 | 70.9 | 69.6 | 67.1 | 66.9 | 68.2 | 68 | 67 | 69.8 | 68 | 66.8 | 67.9 | 100 | 88.1 | 75.5 | 69-70.7 |
| *Phycisphaera mikurensis* | 72.5-74.2 | 73.7 | 73.2 | 71.9 | 71.8 | 72 | 73.2 | 73.5-75.1 | 72.2 | 74.3 | 70.8 | 73.2 | 71.7 | 70.1 | 68.2 | 68.5 | 70.8 | 70.2 | 69.4 | 70.2 | 69.4 | 65.6 | 68 | 88.1 | 100 | 75.6 | 68.4-69.7 |
| *Tepidisphaera mucosa* | 70.8-71.7 | 71 | 71.7 | 71.1 | 70.6 | 73.7 | 74.4 | 72.5-73.7 | 68.6 | 69.6 | 70.1 | 69.9 | 67.1 | 69.1 | 69.1 | 68.8 | 69.7 | 68.1 | 68 | 72.1 | 70.3 | 70 | 72.1 | 75.5 | 75.6 | 100 | 69.2-71 |
